# Pathogenic Variants in Mennonites From Southern Brazil: Implications for Preventive Measures in Public Health

**DOI:** 10.1111/cge.70035

**Published:** 2025-08-05

**Authors:** Luiza Beatriz Mayer de Lima, Eduardo Delabio Auer, Isabela Dall’Oglio Bucco, Valéria Bumiller‐Bini Hoch, Priscila Ianzen dos Santos, Fabiana L. Lopes, Alan Shuldiner, Emilton Lima Júnior, Angelica Beate Winter Boldt

**Affiliations:** ^1^ Postgraduate Program in Internal Medicine Federal University of Paraná (UFPR) Curitiba Brazil; ^2^ Laboratory of Human Molecular Genetics, Department of Genetics Federal University of Paraná (UFPR) Curitiba Brazil; ^3^ Human Genetics Branch National Institute of Mental Health Bethesda Maryland USA; ^4^ Regeneron Genetics Center Tarrytown New York USA

**Keywords:** BTD, FANCM, FLG, founder effect, HFE, Mennonites, monogenic diseases, precision medicine, whole exome sequencing

## Abstract

The Mennonite population has a unique history of 500 years of genetic isolation shaped by at least three demographic bottlenecks, founder effects, inbreeding, epidemics, and migrations. To evaluate their susceptibility for monogenic diseases (MD), we performed whole‐exome sequencing on 325 volunteers from two South Brazilian Mennonite settlements (one urban and another rural). We identified 23 pathogenic variants (P) and 27 likely P, with 22.8% accounting for endocrine, nutritional, and metabolic MDs, 17.5% for developmental anomalies, and 10.5% for nervous system MDs. *HFE* rs1800562 causing hereditary hemochromatosis presented the highest frequency (7.54%), followed by *BTD* rs13078881 for biotinidase deficiency (7.08%), *FLG* rs61816761 for ichthyosis vulgaris and atopic dermatitis (3.38%), and *FANCM* rs147021911 for Fanconi anemia (3.08%). Genomic and genealogical analysis confirmed their European origin, with very low consanguinity and high heterozygosity coefficients, confirming a random selection of refugees that emigrated from widespread settlements in Russia to Brazil in 1930. There was also a slight deviation to Native Americans for self‐reported admixed Mennonites. Even so, founder effects occurred for 96% of P, whose frequencies differed from non‐Finnish Europeans, Amish, and Brazilian populations. These findings highlight the genetic risks in this population, reinforcing the importance of genetic counseling, screening programs, and Personalized and Preventive Medicine strategies to mitigate health risks associated with inherited conditions.

## Graphical abstract

In 325 exomes of South Brazilian Mennonites, we identified 23 pathogenic variants (P) and 27 likely P, with founder effects identified for 96% of P, whose frequencies differed from non‐Finnish Europeans, Amish, and Brazilian populations.

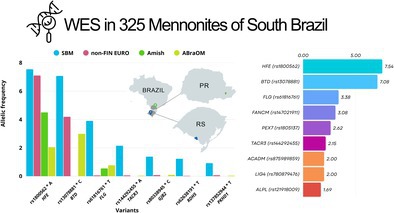

## 1 Introduction

The Mennonites represent the most complex group among Anabaptist populations, which also include the Amish and Hutterites. Anabaptism originated in Switzerland during the Protestant Reformation in the sixteenth century. Characterized by conscious baptism in adulthood, believers faced intense persecution from the Church and the State [1, 2, 3]. Seeking refuge, many Anabaptists migrated to the Netherlands in the 1530s [2]. One particular group, under the leadership of Menno Simons, consolidated their identity as Mennonites, adopting pacifism as their central principle [1, 2]. They subsequently prospered for almost two centuries in Danzig, Prussia (now Gdánsk, Poland), migrating afterwards to the Ukraine between 1787 and 1796, lured by promises from Tsarina Catherine the Great. There, around 2000 Mennonites established prosperous colonies such as Chortitza and Molotschna [3]. However, the Russian Revolution and the impacts of the First World War brought repression, looting, and famine, resulting in large population losses. Between 1923 and 1926, many Mennonites emigrated to Canada, and in 1929, around 6000 sought refuge in South America due to immigration restrictions in North America [3]. In 1930, the first Mennonites arrived in Brazil, settling mainly in the states of Paraná and Santa Catarina. This initial group, made up of approximately 1311 individuals, formed self‐sufficient farming communities, preserving their cultural and religious traditions despite successive migrations [3, 4].

This group has remained strictly separate for five centuries by now, with little gene flow with outsiders [1, 3, 5]. Evolutionarily, the history of Latin American Mennonites is characterized by at least three demographic bottlenecks, inbreeding, epidemics, several migrations due to political and religious persecution, and mass exterminations under the pretext of wars and political conflicts [1, 3, 5]. These evolutionary drift phenomena contribute to the loss and/or fixation of random alleles, reduced genetic diversity, and homozygous revelation of deleterious recessive alleles, causing a higher incidence of genetic diseases [3, 5, 6]. The Mennonite population also maintains well‐documented genealogical records, which facilitate the ascertainment of families and the estimation of population parameters [7]. They therefore become interesting groups for genetic studies and may need precise and targeted medical attention for counseling.

Conservative Mennonites usually adopt traditional health treatments, avoiding modern medicine for reasons of faith. This may lead to late diagnosis, increasing the severity of health problems that could be treated more effectively with modern medical interventions [8]. The lack of epidemiological knowledge regarding the Mennonites leads to care that does not always meet their needs. The lack of public policies aimed at this population further results in worse health indicators for this population [6, 7, 9].

Monogenic diseases (MD) represent around 80% of rare diseases of genetic origin (Online Mendelian Inheritance in Man [OMIM], 2025) [10], but the genetic cause and/or subjacent mechanisms of more than half remain elusive [11]. In part, this is due to the variable expressivity of several MD [11, 12]. Whole exome sequencing (WES) has been a valuable tool in the identification and personalized treatment of MD, considering the mutation profile of a given patient [13, 14]. In this study, we identified pathogenic (P) or likely pathogenic variants (LP) linked with MDs, using WES in the South Brazilian Mennonite (SBM) population. The identification of these variants could help genetic counseling, enable screening, and subsidize Personalized and Preventive Medicine strategies for this population. In addition, the investigation of genetic ancestry can provide complementary information in the interpretation of rare variants, especially in populations under‐represented in genomic databases. We therefore evaluated both ancestry and consanguinity in the SBM, in order to provide a more robust analysis.

## 2 Materials and Methods

### 2.1 Participants and Data Collection

Mennonite representatives, concerned with the consanguinity in the communities, directed a request for the prevention of MDs to the current coordinator of the project (ABWB). This prompted us to discuss the strategy of contact, personal interviews, timing, and location of recruitment with community leaders, and afterwards to invite the population to participate through e‐mails and letters handed out by the residents' associations and through small talks (also in German language), given during community gatherings. Mennonite health workers (physicians, nutritionists, nurses, pharmacists, psychologists), educators, and community leaders were engaged in the project from the very beginning, both to motivate participation in the study and to support the deliverance of general results. The project started in October 2016 with the sampling of anthropometric data and biological material, taken after informed consent from 325 volunteers living in two South‐Brazilian Mennonite communities: 194 (59.7%) in rural Colônia Nova, Aceguá—Rio Grande do Sul (CON) and 131 (40.3%) in urban communities from Curitiba—Paraná (CWB). The sampled group consisted of 55.3% women and 44.7% men with a mean age of 53 years (12.0–95.4) (Figure 2A). Using a questionnaire based on the 2013 Brazilian National Health Survey, we also collected data in personal interviews, with questions about the migratory route of ancestors, eating and living habits, exposure to toxic agents, diagnosis, and family history of complex diseases. Mennonite ethnic origin was evaluated by means of genealogy, surnames, and migratory routes informed in the questionnaire and confirmed using GRanDMA (The Grandmother Database, a genealogical database for Mennonite ancestry) [13], which is a repository of genealogical data extracted from Mennonite church registers and periodicals, dating from the XVII century.

### 2.2 Biological Samples

We collected 8 mL of peripheral blood from each participant in tubes with and without EDTA, after local asepsis. Each participant was identified with a code in order to keep their personal data confidential. DNA was extracted from the peripheral blood mononuclear cells (PBMC) using the Wizard Genomic DNA Purification commercial kit (Promega, Madison, WI, USA).

### 2.3 Exome Sequencing

The exomes were sequenced on the Illumina HiSeq platform at an average depth of 30X. The raw sequencing data was converted to variant call format (VCF) and aligned to the GRCh38/hg38 reference genome. Also, ForestQC software helped with low‐quality variants removal. P and LP variants were classified automatically based on the recommendations of the American College of Medical Genetics (ACMG) [14] for incidental findings in DNA sequencing, on the Varsome website [15]. In addition to incidental variants, variants in genes related to cancer, cardiovascular diseases, dyslipidemia, retinal dystrophies, Fanconi anemia, Lynch syndrome, collagen, and metalloproteinases were also identified by annotating them with the Variant Effect Predictor (VEP)—Ensembl! tool (https://www.ensembl.org/index.html) and classified on the Varsome website (https://varsome.com/) [16, 17]. Finally, all P and LP variants with allele counts lower than four were excluded to minimize the risk of including false‐positive variants resulting from sequencing errors. No exclusion criteria based on kinship were applied, given the endogamous nature of the population studied and the focus of the study on characterizing its genetic structure. Allele frequencies were calculated in the whole sample and after the exclusion of first‐degree relatives, and compared with other populations. These comparisons, as well as the calculation of genomic f consanguinity values and heterozygosity based on surname frequencies, were performed after the exclusion of first‐degree relatives of probands.

### 2.4 Data Collection and Classification

Genetic variant information was gathered from three publicly available databases: OMIM (https://www.omim.org/) [10] ClinVar (https://www.ncbi.nlm.nih.gov/clinvar/) [18], and Varsome (https://varsome.com/) [15] and Franklin (https://franklin.genoox.com) [19]. The OMIM database was used to identify variants associated with specific genetic disorders, providing detailed descriptions of phenotypes and clinical associations. ClinVar was consulted to verify the clinical relevance and categorization of variants in terms of pathogenicity. The Franklin and Varsome databases were used to obtain detailed information on variant classification, including frequency data (based on population studies), protein effect, population data, in silico predictions, functional data, and more.

**FIGURE 1:**
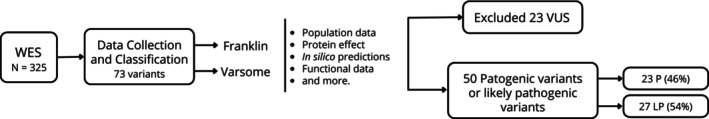
Filtering process of genetic variants, showing the removal of 23 variants of uncertain significance (VUS) and the selection of only pathogenic (P) and likely pathogenic (LP) variants. [Colour figure can be viewed at wileyonlinelibrary.com]

The variants of interest were extracted manually and/or through automated tools provided by the platforms, ensuring the integrity and accuracy of the collected data. Subsequently, the identified variants were classified according to the International Classification of Diseases (ICD) [20], a classification system maintained by the World Health Organization (WHO). This classification facilitated the standardization of disease‐related data and supported the analysis of the variants in a global health context. The data was then analyzed, considering both the clinical implications and the effects at the molecular level (Figure 1).

### 2.5 Ancestry Analysis

For the ancestry analysis, the EthSEQ package in the R 4.2.2 language was used, following the methodology described by Dalfovo and Romanel, 2023 [21]. The genetic database model (SNVs) for reference populations (with known ancestry) was “Gencode.Exome.” This model selected SNVs previously annotated by GENCODE exome in the 1000 Genomes Project data. The reference populations were: European, East Asian, American, South Asian, African, and multiple origins from genotype files in Variant Call Format (VCF). Ancestry and PCA (principal component analysis) variables were inferred and calculated for each individual. The ancestry inference outputted a 3D PCA graph (PCA 1, PCA 2, and PCA 3), also created by the EthSEQ package, to analyze the possible contributions of world populations and a possible admixture/introgression of the Brazilian population. The results were validated by comparing them with historical and genealogical data from the Mennonite population [15], as well as by comparing them with other published genetic studies on populations of similar origin.

### 2.6 Statistical Analysis of the Data

Allele frequencies and counts were calculated and counted using the software Plink 1.9 [22], as well as the *p*‐value for the Hardy–Weinberg equilibrium test (with the mid‐P correction) and genomic inbreeding (*f* coefficient estimates). The allele frequencies of the SBM population were compared to those of different populations using Fisher's exact test. The Mennonite and non‐Finnish European populations were compared to identify variants whose frequencies suffered a possible founder effect in relation to the founder population. The comparison between the Mennonite and Amish populations allows us to identify the effects of genetic drift resulting from 300 years of separation from the original Anabaptist population. The allele frequencies of the non‐FIN EURO and Amish populations were obtained from gnomAD 4.1: https://gnomad.broadinstitute.org/ [23]. The allele frequencies of the Brazilian population were taken from the ABraOM—Arquivo Brasileiro Online de Mutações—website: https://abraom.ib.usp.br/ [24]. The tests were carried out in the R language (version 4.2.2) with multiple testing corrections using the FDR (false discovery rate) method for the number of populations in each variant. All *p*‐values < 0.05 were considered statistically significant.

Before estimating the genomic inbreeding coefficients (*f* coefficients), we conducted a quality control process on the exome data. This included the following steps: (1) Linkage disequilibrium pruning was performed using the command “‐indep‐pairwise 20 000 2000 0.2;” (2) Variants with missing genotype rates exceeding 5% were excluded using “‐geno 0.05;” (3) Variants with a minor allele frequency lower than 5% were also excluded using “‐maf 0.05.” After completing these quality control measures, Plink 1.9 provided the F coefficient estimates for each individual. We then utilized R language (version 4.2.2) to calculate the mean *f* coefficient estimate for the population, along with its standard deviation. We used boxplots and histograms to report individual depth as a quality measure, as well as the number of LP and P variants per individual from the reported variants in this study. These plots were produced using the R language (version 4.2.2) with the “ggplot2” and “vcfR” packages.

Kinship data were obtained through genealogical mapping on the GRanDMA platform [13], using the information collected alongside biological samples. Surname frequencies (of both maternal and paternal origin) were calculated in the general sample and within each set of carriers, given a sample size of more than two individuals.

## 3 Results

### 3.1 Exome Analysis

**FIGURE 2:**
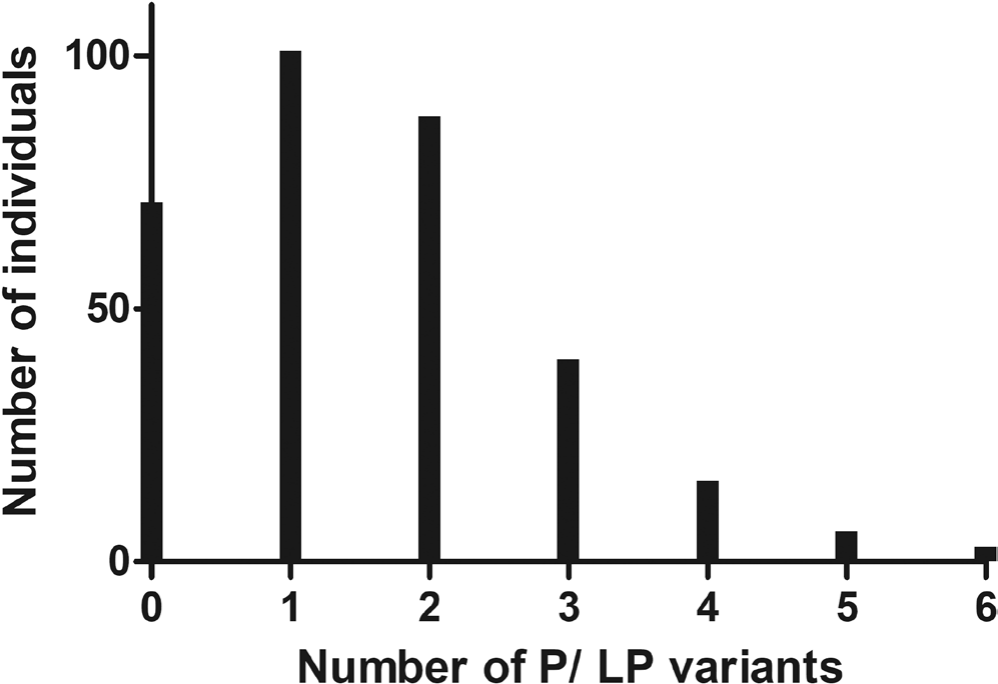
Distribution of the number of pathogenic (P) or probably pathogenic (LP) variants per individual in the Mennonite population of southern Brazil. [Colour figure can be viewed at wileyonlinelibrary.com]

We found 50 P (46%; 23) and/or LP (54%; 27) variants in 49 genes (Tables 1 and S1). Among all P/LP variants, 38 (76%) segregated within families. Individual coverage of each variant is given in Figures S1 and S2 (both for all exomes and only for carriers). The genotype distributions of the variants did not deviate from the Hardy‐Weinberg equilibrium (*p* > 0.05). Among the participants, 78.15% (254) were carriers of P or LP variants. The full distribution of the number of PVs per individual is shown in Figure 2, demonstrating that the majority of individuals (70.5%) carry between 1 and 3 variants.

**TABLE 1:** Rare and low frequency pathogenic variants found in South‐Brazilian Mennonites.

Gene	dbSNP ID	HGVS cDNA	HGVS protein	Type of mutation	SBM %, *n* = 650 (*n =* 278)	Non‐FIN EURO % (*n*)	p*	Amish % (*n*)	p**	ABRAOM % (*n*)	p***
*HFE*	rs1800562	c.845G>A	p.Cys282Tyr	Missense	7.54 *(8.27)*	7.10 (1180000)	0.65 *0.41*	4.50 (912)	**0.015** ***0.033***	2.05 (2342)	***p* < 0.001** ***p < 0.001***
*BTD*	rs13078881	c.1270G>C	p.Asp424His	Missense	7.08 *(6.84)*	4.19 (1180024)	***p* < 0.001** ***0.035***	0.00 (912)	***p* < 0.001** ***p < 0.001***	2.99 (2342)	***p* < 0.001** ***0.0035***
*FLG*	rs61816761	c.1501C>T	p.Arg501Ter	Stop gained	3.38 *(2.88)*	2.14 (1179838)	**0.04** *0.40*	0.55 (912)	***p* < 0.001** ***0.0062***	0.77 (2342)	***p* < 0.001** ***0.0062***
*FANCM*	rs147021911	c.5101C>T	p.Gln1701Ter	Stop gained	3.08 *(2.52)*	0.07 (1179834)	***p* < 0.001** ***p < 0.001***	0.00 (912)	***p* < 0.001** ***p < 0.001***	**—**	**—**
*PEX7*	rs1805137	c.875 T>A	p.Leu292Ter	Stop gained	2.62 *(3.96)*	0.09 (1176024)	***p* < 0.001** ***p < 0.001***	0.00 (912)	***p* < 0.001** ***p < 0.001***	**—**	**—**
*TACR3*	rs144292455	c.824G>A	p.Trp275Ter	Stop gained	2.15 *(2.52)*	0.06 (1179320)	***p* < 0.001** ***p < 0.001***	0.00 (912)	***p* < 0.001** ***p < 0.001***	0.04 (2342)	***p* < 0.001** ***p < 0.001***
*ACADM*	rs875989859	c.347G>A	p.Cys116Tyr	Missense	2.00 *(2.16)*	0.001 (1179986)	***p* < 0.001** ***p < 0.001***	0.00 (912)	***p* < 0.001** ***p < 0.001***	**—**	**—**
*LIG4*	rs780879476	c.613del	p.Ser205LeufsTer29	Frameshift variant	2.00 *(2.16)*	0.009 (1179944)	***p* < 0.001** ***p < 0.001***	0.00 (912)	***p* < 0.001** ***p < 0.001***	**—**	**—**
*ALPL*	rs121918009	c.1001G>A	p.Gly334Asp	Missense	1.69 *(2.16)*	0.0001 (1180032)	***p* < 0.001** ***p < 0.001***	0.00 (910)	***p* < 0.001** ***p < 0.001***	**—**	**—**
*SBDS*	rs113993993	c.258 + 2 T>C	**—**	Splice donor	1.54 *(1.44)*	0.36 (1177666)	***p* < 0.001** ***0.018***	0.00 (910)	***p* < 0.001** ***0.0059***	**—**	**—**
*RAG1*	rs199474678	c.1420C>T	p.Arg474Cys	Missense	1.38 *(0.72)*	0.006 (1180054)	***p* < 0.001** ***p < 0.001***	0.00 (912)	***p* < 0.001** *0.054*	**—**	**—**
*HPS3*	rs121908316	c.1189C>T	p.Arg397Trp	Missense	1.38 *(1.08)*	0.004 (1179892)	***p* < 0.001** ***p < 0.001***	0.00 (912)	***p* < 0.001** ***0.013***	**—**	**—**
*MMP13*	rs797044754	c.772_773insG	p.Asp258GlyfsTer14	Frameshift variant	0.77 *(0.36)*	8.5 × 10^−5^ (1179398)	**0.01** ***p < 0.001***	0.00 (912)	***p* < 0.001** *0.23*		
*RDH5*	rs62638191	c.712G>T	p.Gly238Trp	Missense	1.23 *(2.16)*	0.03 (1179806)	***p* < 0.001** ***p < 0.001***	0.00 (910)	**0.001** ***p < 0.001***	0.17 (2342)	**0.001** ***p < 0.001***
*EIF2B2*	rs113994012	c.599G>T	p.Gly200Val	Missense	0.92 *(0.36)*	0.06 (1178410)	***p* < 0.001** *0.23*	0.00 (912)	**0.0051** *0.23*	**—**	**—**
*HEXA*	rs121907954	c.805G>A	p.Gly269Ser	Missense	0.77 *(0.72)*	0.02 (1180000)	***p* < 0.001** ***0.0018***	0.00 (912)	**0.01** *0.054*	**—**	**—**
*KIZ*	rs587777376	c.52G>T	p.Glu18Ter	Stop gained	1.08 *(0.72)*	0.0006 (1127410)	***p* < 0.001** ***p < 0.001***	0.00 (912)	**0.002** *0.054*	**—**	**—**
*IDUA*	rs199801029	c.979G>C	p.Ala327Pro	Missense	1.08 *(0.36)*	0.02 (1178974)	***p* < 0.001** *0.099*	0.00 (912)	**0.002** *0.23*	**—**	**—**
*PKHD1*	rs137852944	c.107C>T	p.Thr36Met	Missense	0.92 *(0.72)*	0.07 (1176124)	***p* < 0.001** ***0.047***	0.00 (912)	**0.0051** *0.054*	0.04 (2342)	***p* < 0.001** ***0.047***
*COL10A1*	rs779802963	c.211C>T	p.Arg71Ter	Stop gained	0.92 *(0.72)*	0.00 (1179996)	***p* < 0.001** ***p < 0.001***	0.00 (912)	**0.0051** *0.054*	**—**	**—**
*ZMPSTE24*	rs137854889	c.1085dup	p.Leu362PhefsTer19	Frameshift variant	0.62 *(1.08)*	0.05 (1170512)	***p* < 0.001** ***p < 0.001***	0.00 (910)	**0.03** ***0.013***	**—**	**—**
*FBN1*	rs794728200	c.3020 T>G	p.Leu1007Arg	Missense	0.62 *(0.72)*	0.0004 (1180038)	***p* < 0.001** ***p < 0.001***	0.00 (912)	**0.03** *0.054*	**—**	**—**
*CYBA*	rs779809359	c.261C>G	p.Tyr87Ter	Stop gained	0.62 *(0.36)*	**—**	**—**	**—**	**—**	**—**	**—**

*Note*: Values in italics were obtained after the exclusion of first‐degree relatives. FirstSBM % (*n*)**—**Percentage of allele frequencies found in South‐Brazilian Mennonites and sample number of alleles; “**—**” not applicable; non‐FIN EURO % (*n*)**—**allele frequencies and number of alleles reported in the gnomAD database (v4.1, non‐Finnish European populations); p*— *p*‐value calculated with Fisher's exact test to compare allele frequencies between South Brazilian Mennonites versus non‐Finnish Europeans; Amish % (*n*)**—**allele frequencies and number of alleles reported in the gnomAD database (v4.1, Amish population); p**— *p*‐value calculated Fisher's exact test to compare allele frequency between South Brazilian Mennonites and Amish populations; ABraOM % (*n*)—allele frequencies and number of alleles reported in ABraOM (database of Brazilian genomic variants), p***— *p*‐value calculated with Fisher's exact test to compare allele frequencies between South Brazilian Mennonites and Brazilians; all *p*‐values < 0.05 were considered statistically significant; In bold: statistically significant *p*‐values; dbSNP ID—identifier assigned to a single nucleotide polymorphism (SNP) in the database of single nucleotide polymorphisms; HGVS cDNA—human genome variation society notation for variants at the complementary DNA (cDNA) level; HGVS protein—human genome variation society notation for variants at the protein level. *HFE* (homeostatic iron regulator); *BTD* (biotinidase); *FLG* (Filaggrin); *FANCM* (Fanconi Anemia Complementation Group M); *GALNS* (Galactosamine *N*‐Acetyl‐6‐Sulfatase); *PEX7* (Peroxisomal Biogenesis Factor 7); *ACADM* (Acyl‐CoA Dehydrogenase Medium Chain); *LIG4* (DNA Ligase 4); *TACR3* (Tachykinin Receptor 3); *ALPL* (Alkaline Phosphatase, Biomineralization Associated); *C8A* (Complement Component 8 Alpha Chain); *RAG1* (Recombination Activating 1); *GJB2* (Gap Junction Protein Beta 2, Connexin 26); *RDH5* (Retinol Dehydrogenase 5); *EIF2B2* (Eukaryotic Translation Initiation Factor 2B Subunit Beta); *HEXA* (Hexosaminidase Subunit Alpha); *LONP1* (Lon Peptidase 1, Mitochondrial); *HIBCH* (3‐Hydroxyisobutyryl‐CoA Hydrolase); *KIZ* (Kizuna Centrosomal Protein); *IDUA* (Alpha‐L‐Iduronidase); *KIZ* (Kizuna Centrosomal Protein) *PKHD1* (PKHD1 Fibrocystin/Polyductin); *COL10A1* (Collagen Type X Alpha 1 Chain); *ZMPSTE24* (Zinc Metallopeptidase STE24); *FBN1* (Fibrillin 1); *CYBA* (Cytochrome B‐245 Alpha Chain); *VCP* (Valosin Containing Protein).

**FIGURE 3:**
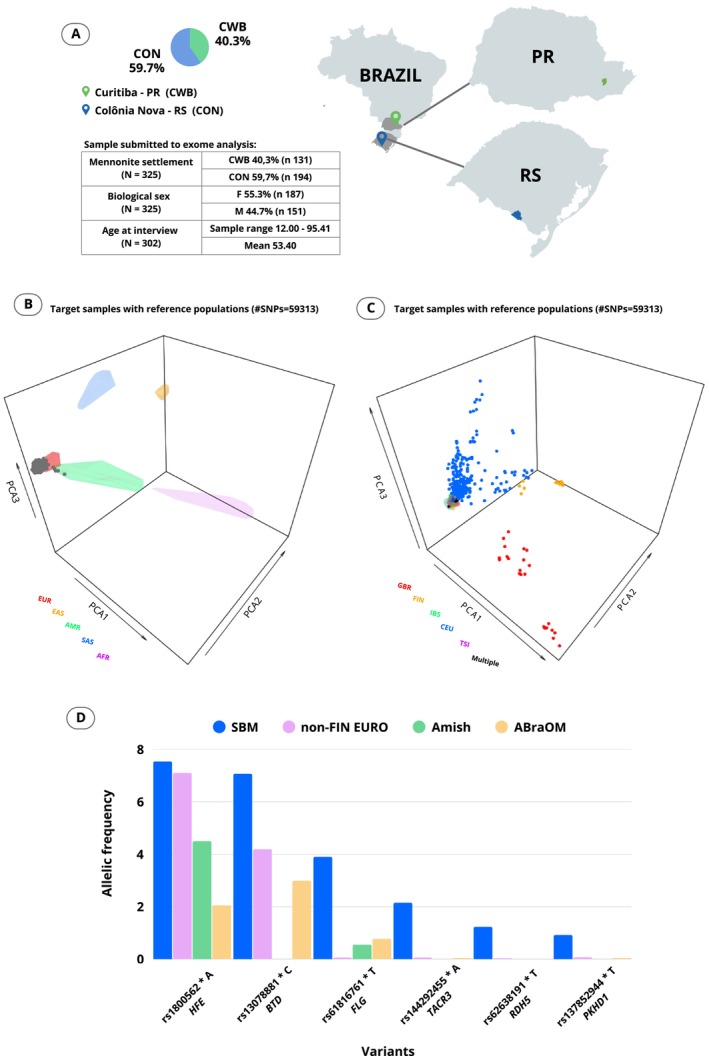
Sampling and variant classification. (A) Map of Brazil highlighting the states of Paraná (PR) and Rio Grande do Sul (RS), where the South Brazilian Mennonite colonies are located. It also shows the proportion of samples per location and a table describing them. (B) Principal component analysis (PCA) graph comparing target samples with reference populations, based on 59 313 exons SNPs. The single gray points represent individuals from the Mennonite population, while convex hulls display colors representing the location of reference population groups from the 1000 genomes project in PCA plot. Each color represents a different population group: EUR (European)—red; EAS (East Asian)—yellow; AMR (American—native and mixed)—green; SAS (South Asian)—blue and AFR (African)—purple. (C) Principal component analysis (PCA) graph comparing target samples with European reference populations, based on 59 313 exon SNPs. The single points represent individuals from the Mennonite population and the color of each target individual point is set according to the estimated ancestry and refers to the corresponding reference ancestry color, while the convex hulls display colors representing the location of reference population groups from the 1000 genomes project in PCA plot. The colors represent different populations: GBR (England and Scotland)—red; FIN (Finland)—yellow; IBS (Spain)—green; CEU (Utah, USA, with northern and western European ancestry)—blue; TSI (Tuscany, Italy)—purple; and multiple (diverse origins)—black. (D) Differences in the allelic frequency of the variants in the Southern Brazilian Mennonite (SBM), non‐Finnish, Amish and Brazilian population, based on data from gnomAD 4.1 and the ABraOM.

Basic data on population sampling are given in Figure 3A, clustering with Europeans (Figure 3B), especially with the Utah European‐derived population (Figure 3C—see section “ancestry analysis” for further details). The allele frequency of 22 variants differed from that of non‐Finnish Europeans, 23 differed from the Amish population, and 6 differed from the Brazilian population (Figure 3D). Even after the exclusion of first‐degree relatives (reducing sample size to 139 individuals), the frequencies of all but four gene variants remained significantly different from non‐Finnish Europeans, reflecting a founder effect (Table 1).

The variants with the highest allele frequencies included: rs1800562 (c.845G>A; p.Cys282Tyr) in the *HFE* gene (7.54%), associated with hereditary hemochromatosis; rs13078881 (c.1270G>C; p.Asp424His) in the *BTD* gene (7.08%), linked to biotinidase deficiency; rs61816761 (c.1501C>T; p.Arg501Ter) in the *FLG* gene (3.38%), associated with ichthyosis vulgaris and atopic dermatitis type 2; and rs147021911 (c.5101C>T; p.Gln1701Ter) in the *FANCM* gene (3.08%), related to cancer susceptibility, among others (Table 1). According to the ICD‐11 performed for each variant based on phenotypic data collected from the OMIM, ClinVar, and Franklin databases, 20.7% of the variants lead to dysfunctions related to endocrine, nutritional, and metabolic diseases, followed by developmental anomalies (15.5%) and diseases of the nervous system (10.3). Among the genetic conditions related to these variants, 62% (31) are related to autosomal recessive (AR) traits, 28% (14) to autosomal dominant (AD) traits, 6% (3) are related to both, and 4% (2) do not have enough data to report. According to the mutation type classification, 44% (22) of the variants were missense, followed by 20% (10) stop‐gained, 12% (6) splice acceptor, 12% (6) frameshift, 6% (3) splice donor, and 6% (3) missense in the splice region.

### 3.2 Ancestry Analysis

The distribution of the Mennonite samples (Figure 3B) strongly grouped with the European population. There is a slight dispersion towards the Native American population, indicating the presence of 9.23% (*n* = 30) mixed‐ancestry individuals. All of them reported at least one parent or grandparent with a non‐Mennonite German surname. In addition, four reported a Portuguese surname. Except for these four, none of them reported admixture with Native Americans. Unmixed SBM grouped with European reference populations: the majority with individuals of northern and western European origin (CEU from Utah, USA), followed by individuals from both England and Scotland (GBR) and Finland (FIN) (Figure 3C). This genetic distribution is consistent with the migratory history of the Mennonites, who moved from Europe to different regions over the centuries, including South America.

### 3.3 Consanguinity and Heterozygosity Analysis

**FIGURE 4:**
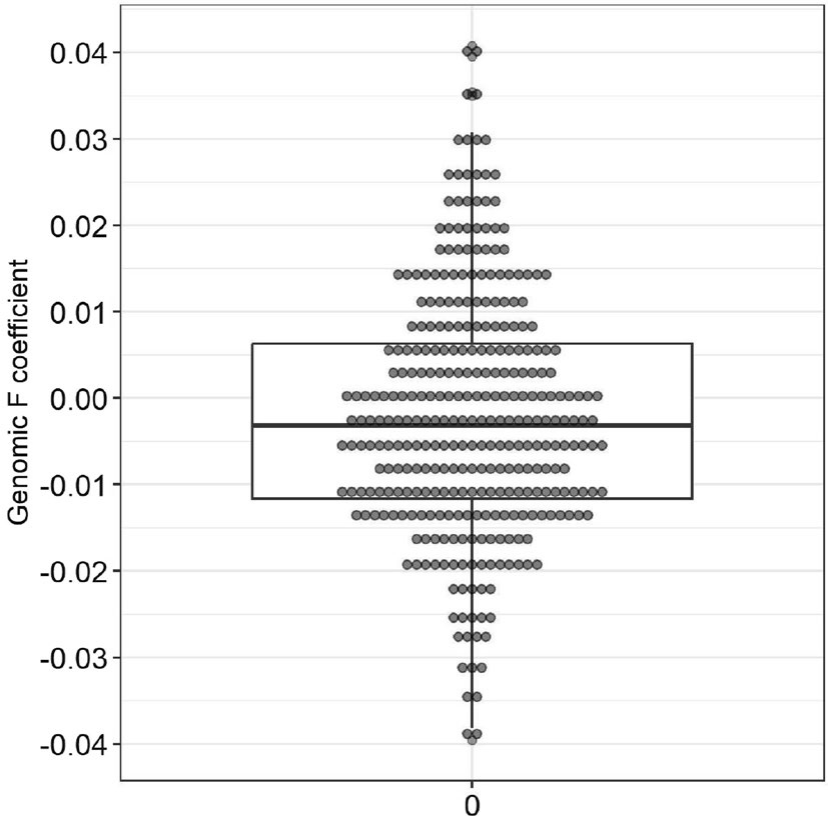
Distribution of the inbreeding coefficient (f) in the Mennonite population analyzed (*n* = 325).

The average genomic inbreeding coefficient (*f*) in the Mennonite population analyzed (325 individuals with exomes) was −0.002, with a standard deviation of 0.01, indicating a low level of inbreeding (Figure 4). Among the carriers of the five most frequent P variants, *f* values indicated a very low level of inbreeding (Table 2). We also evaluated surname diversity as a measure of heterozygosity, taking each surname (of maternal and paternal origin) as an allele. We found 86 different surnames, corresponding to 97.7% of expected heterozygosity within our subsample excluding first‐degree relatives (*n* = 139). Among the surnames, 15% were of non‐Mennonite origin (all but three, belonging to German immigrants who arrived in South Brazil during the XIX century). This figure was quite similar to the values calculated within each set of allele carriers, which ranged from 97% to 78% (Table S2), being positively correlated to the number of allele carriers (Spearman *r* = 0.78, *p* < 0.0001). These results confirm the historical reports of the exodus from Russia, according to which at least half of the Brazilian Mennonite pioneers in 1930 derived from different Mennonite settlements distributed from Ukraine to Siberia 4.

**TABLE 2:** Frequency and distribution of the five most common pathogenic variants identified in first‐degree unrelated South Brazilian Mennonites (*N* = 139).

Variant	Gene	Location CON CWB, *n* (%)		Number of carriers (%)	Heterozygosity by surname analysis (±SD)	*f* coefficient of consanguinity (SD)
rs1800562	*HFE*	*n* = 11 (47.8)	*n* = 12 (52.2)	*n* = 23 (16.55)	95.3 ± 0.2	*n* = 23 0.00 (±0.01)
rs13078881	*BTD*	*n* = 11 (57.9)	*n* = 8 (42.1)	*n* = 19 (13.67)	95.4 ± 0.2	*n* = 19 0.00 (±0.01)
rs61816761	*FLG*	*n* = 3 (37.5)	*n* = 5 (62.5)	*n* = 8 (5.76)	92.2 ± 0.4	*n* = 8 0.00 (±0.02)
rs147021911	*FANCM*	*n* = 3 (42.9)	*n* = 4 (57.1)	*n* = 7 (5.04)	90.8 ± 0.6	*n* = 7 −0.01 (±0.01)
rs1805137	*PEX7*	*n* = 6 (54.5)	*n* = 5 (45.5)	*n* = 11 (7.91)	94.2 ± 0.2	*n* = 11 0.00 (±0.01)

*Note*: The table highlights the number of carriers, the heterozygosity by the recurrence of surnames, and the average genomic consanguinity coefficient (*f*), providing insights into the origin and spread of these variants in the South Brazilian Mennonite population. All results were calculated after the exclusion of 1st degree relatives (total sample size = 139 individuals).

Abbreviations: BTD, Biotinidase; CON, Colônia Nova, in Aceguá—Rio Grande do Sul (RS); CWB, Curitiba—Paraná (PR); FANCM, Fanconi anemia complementation group M; FLG, filaggrin; HFE, homeostatic iron regulator; PEX7, peroxisomal biogenesis factor 7; SD, standard deviation.

## 4 Discussion

The 325 sequenced SBM showed predominantly northern and western European ancestry, historically corroborating the origin of the Mennonites in the Netherlands in the mid‐1530s and reflecting migratory patterns and low miscegenation over the generations [1, 2]. We also found a clear founder effect for most variants, even after exclusion of first‐degree relatives. This result reflects ancient kinship ties and is related to demographic events that restricted gene flow over 25 generations [1, 3, 5]. Mennonites traditionally encourage marriages within the group, in addition to having an agrarian and cohesive lifestyle, which, in practice, restricted miscegenation with external populations. This isolation was reinforced by the Russian tsarist government, which required Mennonite settlements to be kept apart from other Catholic or Protestant settlements of German or Russian villages, in order to prevent any conflicts [4].

However, unlike Argentinean Old Order Mennonites, who showed no evidence of admixture [25], 30 SBM individuals presented evidence for some degree of gene flow with Native Americans. All of them reported at least one parent or grandparent with a non‐Mennonite surname of German origin, with only four exceptions (surname of Portuguese or Polish origin). This reflects the cultural identification of Mennonites with the fourth generation of Brazilians presenting German origin, who settled in the Itajaí valley (South Brazil) in the middle of the nineteeth century. In contrast with the Mennonites, German immigrants were not organized in a closed community, slowly permitting gene flow from Brazilians of major Portuguese descent miscegenated with the original peoples of the major South‐Brazilian Guarani and Kaingang tribes, as well as with the descendants of Africans from Angola and Mozambique, brought as slaves in the former centuries [3, 4].

The inbreeding coefficient (*f*) based on the exome data was surprisingly low. In fact, no kinship relationship was identified between the Mennonite parents of the only two P variant homozygotes, neither for the *HFE* gene nor for the *BTD* gene. These data suggest that the formation of Mennonite colonies in Brazil occurred predominantly through unrelated individuals, and agrees with the school records of Gnadental, one of the first Mennonite settlements in Brazil, which reported around 46% of the families (17 out of a total of 37, made up of 5 to 6 members each) coming from different Russian settlements [4]. As a result, the effective population size (*N*_*e*_) in the 1940s was estimated at around 154 individuals, corresponding to approximately 75 couples, most of whom came from different colonies in Russia and had no close kinship ties. This pattern of population establishment may have contributed to the low prevalence of homozygotes and consequently of MD among SBM. This finding also suggests that the P and LP variants present in the population have a more remote origin, prior to migration to Brazil.

The high frequency of P/LP variants in SBM is consistent with findings in other Mennonite populations, where genetic continuity and founder effects have played a significant role in shaping the genetic landscape [3, 5]. It is interesting to note that the rs121918009 variant in *ALPL* (c.1001G>A; p.Gly334Asp) (OMIM*171760; ICD‐11 5C64.3) found in SBM with an allele frequency of 1.69% (Table 2), and associated with hypophosphatasia, has been reported in the Canadian Mennonite population in patients with hypophosphatasia, described as p.Gly317Asp (the same codon but after discounting the signal peptide) [26]. On the other hand, some variants previously described in Mennonite communities were not identified in our sequenced sample or did not meet the inclusion criteria adopted, which requires the presence of at least four alleles to ensure greater robustness of the findings and minimize the occurrence of false positives. These variants include: *TBX22* (c.359G>T; p.Gly118Cys), *CYP17* (c.1434‐1437dupCATC; p.Pro480Hisfs Ter27), *CFTR* (c.1521‐1523delCTT; p.F508del) and *RYR1* (c.1840C>T; p.Arg6124Cys), among others [3, 5]. The presence or absence of certain genetic variants in Mennonite communities in the north of the USA versus the SBM can be explained by the difference in the origin of the founders, and the geographical and historical separation between the groups.

The frequencies of all P variants differed from those reported in non‐Finnish European populations and from the Amish population, except for the *HFE* rs1800562 variant, which showed a frequency similar to that of non‐Finnish European populations. These findings highlight the impact of genetic drift and founder effects resulting from at least three demographic bottlenecks, which set the Mennonites apart from their ancestral European groups (such as the Swiss, German, Belgian, Dutch, and others) as well as from a separate branch that led to the emergence of the Amish approximately three centuries ago [3]. Furthermore, most of the variants (86% or 43/50) were absent from the ABraOM database. This database showcases the genomic diversity of admixed elderly individuals sampled in São Paulo city (Southeast Brazil). Thus, it does not fully represent the broader Brazilian population, and the difference in allele frequency between the populations may be even greater. Moreover, the absence of P/LP variants in this database may be explained by their association with reduced longevity [24].

This study did not include a detailed clinical analysis of the individuals carrying the variants identified, but represents a crucial starting point for future research, especially in the context of preventive and predictive medicine. Among the variants, *HFE* rs1800562 (c.845G>A; p.C282Y) is the most prevalent cause of Haemochromatosis type 1H (HR, OMIM*613609; ICD‐11 5C64.0), an autosomal recessive disorder characterized by excessive iron accumulation. This mutation disrupts a critical disulfide bond in the HFE protein by deleting a cysteine at position 282, impairing its ability to bind, transport, and present beta‐2‐microglobulin on the cell surface [21]. The disease is often underdiagnosed due to incomplete biochemical penetrance (75% in men, 50% in women) and variable expressivity, with symptoms usually appearing in middle age and being milder in women due to menstrual blood loss [27, 28]. Its allele frequency is the least altered by drift and founding effect, most probably due to its high frequency in Europeans. Another example is the rs13078881 variant in the *BTD* gene, linked to biotinidase deficiency (OMIM*609019; ICD‐11 4B4Y), a vitamin metabolism disorder. This variant reduces biotinidase activity by approximately 50%, impairing biotin recycling [28, 29]. Western blot assays and enzyme activity for this allele vary between individuals, but it is generally accepted that it reduces enzyme activity by around 50% [30]. Individuals with profound deficiency (< 10% of normal activity) usually show symptoms such as optic atrophy, hypotonia, seizures, hair loss, and skin rashes, often appearing in early childhood [29]. Partial deficiency may only manifest under stress, such as during infections, with symptoms including neurological, dermatological, and visual disorders [29, 30]. Early biotin supplementation can effectively prevent or control these symptoms, highlighting the importance of early detection [30]. (Table S3—BTD rs13078881 references). The rs61816761 variant in the *FLG* gene (c.1501C>T; p.Arg501Ter) (OMIM*135940; ICD‐11 EB00.2) is a stop‐gain mutation that leads to premature termination of the filaggrin protein, commonly associated with atopic dermatitis and ichthyosis vulgaris. Homozygous or compound heterozygous individuals have more severe forms of ichthyosis vulgaris compared to heterozygotes, with the variant segregating in autosomal recessive and dominant patterns over generations. A summary of all variants and associated phenotypes is given in Table S3.

Regarding the limitations of this study, WES is considered a powerful and easily accessible method for genetic diseases in terms of rapid and economical diagnostic yield. It has been observed that its performance is only 2% lower than that of whole genome sequencing (WGS) tests. However, it does not report non‐coding alleles that regulate gene expression [31, 32, 33]. Although we did not confirm the variants by Sanger sequencing, they were shared among relatives, confirming their existence. Moreover, the Mennonite population in South Brazil is much larger, and the small sample size must be taken into consideration to avoid overinterpretation of the data. Furthermore, although there are clear phenotypic correlations for some variants, many others remain ambiguous regarding their clinical impact due to insufficient documentation or due to the wide variability of genetic and environmental factors [12]. Studies that identified new disease‐associated variants through WES in Old Order Amish and Mennonite (Plain) populations in Pennsylvania highlight the importance of further research into these genetic mechanisms and emphasize the need to integrate genomic approaches into medical practice, taking into account the regional particularities and clinical specificities of these communities [34]. This highlights the potential for discovering new genotype–phenotype relationships within the SBM population.

Personalized reference panels, such as those developed for North‐American Anabaptist populations, have been shown to be effective in enabling genetic studies and improving the detection of variants in isolated groups [7]. Although thousands of Mennonites live in Central and South America, their genetic and epidemiological profile is still poorly known and cannot be assumed to be the same as their North American relatives. This was underscored in this study by the genetic differences of SBM and the Amish population. Inclusive policies that respect the cultural and regional specificities of the Mennonite population are crucial to addressing their public health needs and improving the well‐being of these historically marginalized communities. We further hope to build an analogous panel to improve the early identification of P variants in SBMs. Furthermore, case studies focusing on predictive, preventive, personalized, and participatory medicine would be valuable to enhance the understanding of these variants and provide more effective care for affected individuals [31]. Considering that most genetic variants classified as P/LP are associated with recessive MDs, moderate consanguinity among Mennonites increases the frequency of heterozygous carriers; consequently, it raises the risk of couples having children affected by these conditions [33]. Given this scenario, strategies to disseminate the findings of this study to healthcare professionals, offer genetic counseling, expand access to genetic testing, and provide regular medical follow‐up become essential measures to mitigate the clinical impacts of these genetic variants in the SBM population, aiming to improve the quality of life for affected families.

## Ethics Statement

Sample collection and exome sequencing were approved by the National Committee of Ethics in Research (CONEP) through CAAE n. 40798315.1.0000.5263 (protocol 1215264) and the Ethics Committee of Human Research from the Health Sciences Sector of the Federal University of Paraná (CEP SCS‐UFPR), approved under the CAAE ethics review certificate 55297916.6.0000.0102, (protocol 1.545.447 on 16.05.2016, with amendments approved as 2.204.113 on 07.04.2017 and 4.067.744 on 03.06.2020).

## Conflicts of Interest

The authors declare no conflicts of interest.

## Supporting information


**Figure S1:** Supporting Information.

**Figure S2:** Supporting Information.

**Data S1:** Supporting Information.

## Data Availability

The data that support the findings of this study are available on request from the corresponding author. The data are not publicly available due to privacy or ethical restrictions.

## References

[1] H. Penner , H. Gerlach , and H. Quiring , Weltweite Bruderschaft. Ein Mennonitisches Geschichtsbuch (Bücher, 1984).

[2] J. Urry , “Mennonites, Anthropology, and History: A Complicated Intellectual Relationship,” Journal of Mennonite Studies 39 (2021): 15–41, accessed October 8, 2024, https://jms.uwinnipeg.ca/index.php/jms/article/view/2093.

[3] F. L. Lopes , L. Hou , A. B. W. Boldt , et al., “Finding Rare, Disease‐Associated Variants in Isolated Groups: Potential Advantages of Mennonite Populations,” Human Biology 88, no. 2 (2016): 109–120, 10.13110/humanbiology.88.2.0109. 28162000

[4] P. P. Klassen , Die rußlanddeutschen Mennoniten in Brasilien: Witmarsum am Alto Rio Krauel und Auhagen auf dem Stoltz‐Plateau in Santa Catarina (Mennonitischer Geschichtsverein, 1995).

[5] N. C. Orton , A. M. Innes , A. E. Chudley , and N. T. Bech‐Hansen , “Unique Disease Heritage of the Dutch‐German Mennonite Population,” American Journal of Medical Genetics. Part A 146A, no. 8 (2008): 1072–1087, 10.1002/ajmg.a.32061. 18348259

[6] L. C. Oliveira , A. C. Dornelles , R. M. Nisihara , et al., “The Second Highest Prevalence of Celiac Disease Worldwide: Genetic and Metabolic Insights in Southern Brazilian Mennonites,” Genes 14, no. 5 (2023): 1026, 10.3390/genes14051026. 37239386 PMC10218569

[7] L. Hou , R. L. Kember , J. C. Roach , et al., “A Population‐Specific Reference Panel Empowers Genetic Studies of Anabaptist Populations,” Scientific Reports 7, no. 1 (2017): 6079, 10.1038/s41598-017-05445-3. 28729679 PMC5519631

[8] M. Hiebert and I. Hiebert , Erzählungen von mennonitischen Frauen aus Bolivien. (Imago Mundi, Santa Cruz, 2017).

[9] M. C. Boldt , L. C. Oliveira , G. C. Kretzschmar , et al., “Depression and Health Self‐Perception: Associations Within the Isolated Mennonite Population in South Brazil,” Journal of Immigrant and Minority Health 22, no. 6 (2020): 1265–1272, 10.1007/s10903-020-01046-x. 32729102

[10] McKusick‐Nathans Institute of Genetic Medicine, Johns Hopkins University (Baltimore, MD) , Início ‐ OMIM, (McKusick‐Nathans Institute of Genetic Medicine, Johns Hopkins University (Baltimore, MD), 2024. accessed September 23, 2024), https://www.omim.org/.

[11] K. M. T. H. Rahit and M. Tarailo‐Graovac , “Genetic Modifiers and Rare Mendelian Disease,” Genes (Basel) 11, no. 3 (2020): 239, 10.3390/genes11030239. 32106447 PMC7140819

[12] D. N. Cooper , M. Krawczak , C. Polychronakos , C. Tyler‐Smith , and H. Kehrer‐Sawatzki , “Where Genotype Is Not Predictive of Phenotype: Towards an Understanding of the Molecular Basis of Reduced Penetrance in Human Inherited Disease,” Human Genetics 132, no. 10 (2013): 1077–1130, 10.1007/s00439-013-1331-2. 23820649 PMC3778950

[13] “GMOL | Home of GRanDMA OnLine,” 2025, accessed February 16, 2025, https://grandmaonline.org/gmol‐7/loginConfirmation.asp.

[14] S. Richards , N. Aziz , S. Bale , et al., “Standards and Guidelines for the Interpretation of Sequence Variants: A Joint Consensus Recommendation of the American College of Medical Genetics and Genomics and the Association for Molecular Pathology,” Genetics in Medicine 17, no. 5 (2015): 405–424, 10.1038/gim.2015.30. 25741868 PMC4544753

[15] C. Kopanos , V. Tsiolkas , A. Kouris , et al., “VarSome: The Human Genomic Variant Search Engine,” Bioinformatics 35, no. 11 (2019): 1978–1980, 10.1093/bioinformatics/bty897. 30376034 PMC6546127

[16] W. McLaren , L. Gil , S. E. Hunt , et al., “The Ensembl Variant Effect Predictor,” Genome Biology 17, no. 1 (2016): 122, 10.1186/s13059-016-0974-4. 27268795 PMC4893825

[17] A. D. Yates , P. Achuthan , W. Akanni , et al., “Ensembl 2020,” Nucleic Acids Research 48, no. D1 (2020): D682–D688, 10.1093/nar/gkz966. 31691826 PMC7145704

[18] M. J. Landrum , J. M. Lee , G. R. Riley , et al., “ClinVar: Public Archive of Relationships Among Sequence Variation and Human Phenotype,” Nucleic Acids Research 42, no. Database issue (2014): D980–D985, 10.1093/nar/gkt1113. 24234437 PMC3965032

[19] “Franklin by Genoox,” accessed November 27, 2024, https://franklin.genoox.com/clinical‐db/home.

[20] “International Classification of Diseases (ICD),” 2022, accessed September 23, 2024, https://www.who.int/standards/classifications/classification‐of‐diseases.

[21] D. Dalfovo and A. Romanel , “Analysis of Genetic Ancestry from NGS Data Using EthSEQ,” Curr Protocols 3, no. 2 (2023): e663, 10.1002/cpz1.663. 36779822

[22] C. C. Chang , C. C. Chow , L. C. Tellier , S. Vattikuti , S. M. Purcell , and J. J. Lee , “Second‐Generation PLINK: Rising to the Challenge of Larger and Richer Datasets,” GigaScience 4, no. 1 (2015): 7, 10.1186/s13742-015-0047-8. 25722852 PMC4342193

[23] S. Gudmundsson , M. Singer‐Berk , N. A. Watts , et al., “Variant Interpretation Using Population Databases: Lessons From gnomAD,” Human Mutation 43, no. 8 (2022): 1012–1030, 10.1002/humu.24309. 34859531 PMC9160216

[24] “ABraOM: Brazilian Genomic Variants,” 2025, accessed February 18, 2025, https://abraom.ib.usp.br/search.php.

[25] J. Pardo‐Seco , C. Llull , G. Berardi , et al., “Genomic Continuity of Argentinean Mennonites,” Scientific Reports 6 (2016): 36392, 10.1038/srep36392. 27824108 PMC5099698

[26] C. R. Greenberg , C. L. Taylor , J. C. Haworth , et al., “A Homoallelic Gly317‐>Asp Mutation in ALPL Causes the Perinatal (Lethal) Form of Hypophosphatasia in Canadian Mennonites,” Genomics 17, no. 1 (1993): 215–217, 10.1006/geno.1993.1305. 8406453

[27] C. A. Molina , N. G. Ros , R. G. Tarancón , L. R. Varas , V. R. Flores , and S. I. Álvarez , “Hereditary Hemochromatosis: An Update Vision of the Laboratory Diagnosis,” Journal of Trace Elements in Medicine and Biology 78 (2023): 127194, 10.1016/j.jtemb.2023.127194. 37163822

[28] E. T. Strovel , T. M. Cowan , A. I. Scott , and B. Wolf , “Laboratory Diagnosis of Biotinidase Deficiency, 2017 Update: A Technical Standard and Guideline of the American College of Medical Genetics and Genomics,” Genetics in Medicine 19, no. 10 (2017): 1–10, 10.1038/gim.2017.84. 28682309

[29] K. L. Swango , M. Demirkol , G. Hüner , et al., “Partial Biotinidase Deficiency Is Usually due to the D444H Mutation in the Biotinidase Gene,” Human Genetics 102, no. 5 (1998): 571–575, 10.1007/s004390050742. 9654207

[30] T. Borsatto , F. Sperb‐Ludwig , H. J. Blom , and I. V. D. Schwartz , “Effect of *BTD* Gene Variants on *in Vitro* Biotinidase Activity,” Molecular Genetics and Metabolism 127, no. 4 (2019): 361–367, 10.1016/j.ymgme.2019.07.006. 31337602

[31] O. S. Glotov , A. N. Chernov , and A. S. Glotov , “Human Exome Sequencing and Prospects for Predictive Medicine: Analysis of International Data and Own Experience,” Journal of Personalized Medicine 13, no. 8 (2023): 1236, 10.3390/jpm13081236. 37623486 PMC10455459

[32] P. Udupa and D. K. Ghosh , “Implementation of Exome Sequencing to Identify Rare Genetic Diseases,” in Reverse Engineering of Regulatory Networks, ed. S. Mandal (Springer US, 2024), 79–98, 10.1007/978-1-0716-3461-5_5. 37803113

[33] Y. Zhang and Z. Y. Wu , “Gene Therapy for Monogenic Disorders: Challenges, Strategies, and Perspectives,” Journal of Genetics and Genomics 51, no. 2 (2024): 133–143, 10.1016/j.jgg.2023.08.001. 37586590

[34] E. G. Puffenberger , R. N. Jinks , C. Sougnez , et al., “Genetic Mapping and Exome Sequencing Identify Variants Associated With Five Novel Diseases,” PLoS One 7, no. 1 (2012): e28936, 10.1371/journal.pone.0028936. 22279524 PMC3260153

