# Supplementary figures and images for: Pathogenic Variants in Mennonites From Southern Brazil: Implications for Preventive Measures in Public Health
Source: Clin Genet. 2025 Aug 5;109(2):266–76. doi: 10.1111/cge.70035 (PMC12779222; doi:10.1111/cge.70035)

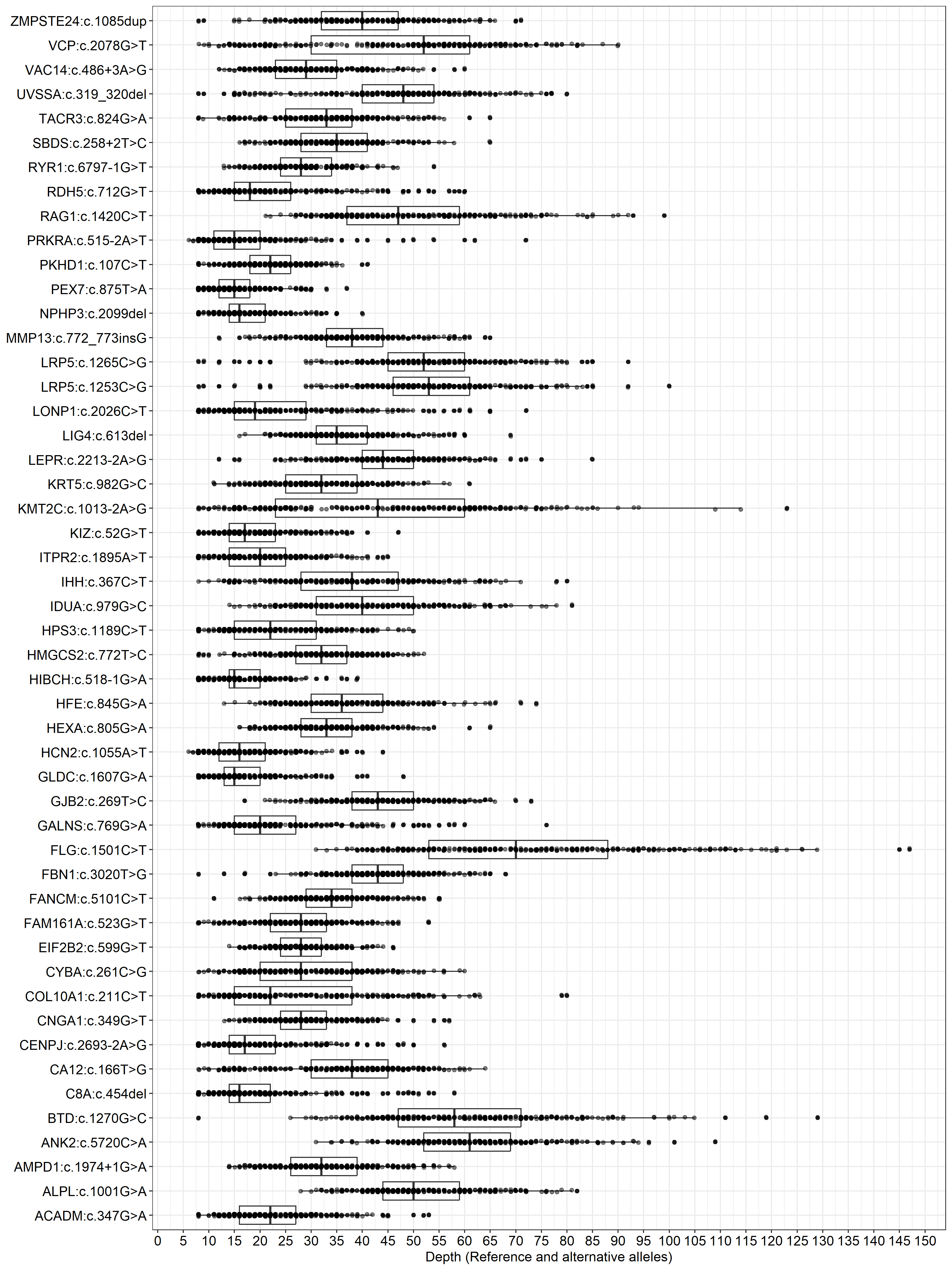

Supplement: Supplementary file 1 — Figure S1: Supporting Information. [file CGE-109-266-s001.tiff]

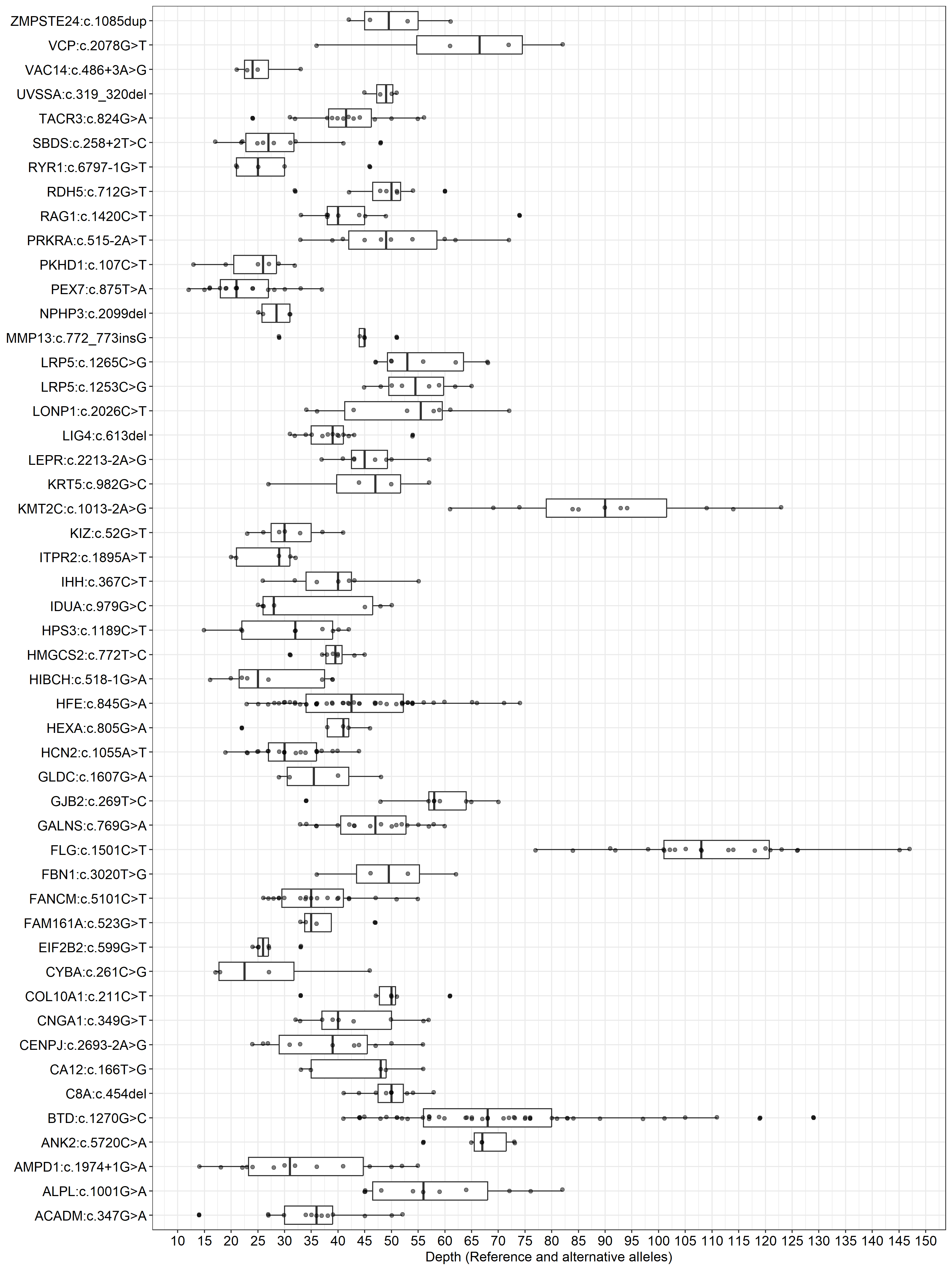

Supplement: Supplementary file 2 — Figure S2: Supporting Information. [file CGE-109-266-s003.tiff]
