# Supplementary material for: Pathogenic Variants in Mennonites From Southern Brazil: Implications for Preventive Measures in Public Health
Source: Clin Genet. 2025 Aug 5;109(2):266–76. doi: 10.1111/cge.70035 (PMC12779222; doi:10.1111/cge.70035)
Supplement: Supplementary file 3 — Data S1: Supporting Information. [file CGE-109-266-s002.docx]

**Supplementary material**

**Supplementary Figures**

Supplementary Figure 1. Sequencing depth of all included exomes, for pathogenic/ likely pathogenic variants.

Pathogenic alleles were found in the following genes: *HFE* (Homeostatic iron regulator); *BTD* (Biotinidase); *FLG* (Filaggrin); *FANCM* (Fanconi Anemia Complementation Group M); *GALNS* (Galactosamine N-Acetyl-6-Sulfatase); *PEX7* (Peroxisomal Biogenesis Factor 7); *ACADM* (Acyl-CoA Dehydrogenase Medium Chain); *LIG4* (DNA Ligase 4); *TACR3* (Tachykinin Receptor 3); *ALPL* (Alkaline Phosphatase, Biomineralization Associated); *C8A* (Complement Component 8 Alpha Chain); *RAG1* (Recombination Activating 1); *GJB2* (Gap Junction Protein Beta 2, Connexin 26); *RDH5* (Retinol Dehydrogenase 5); *EIF2B2* (Eukaryotic Translation Initiation Factor 2B Subunit Beta); *HEXA* (Hexosaminidase Subunit Alpha); *LONP1* (Lon Peptidase 1, Mitochondrial); *HIBCH* (3-Hydroxyisobutyryl-CoA Hydrolase); *KIZ* (Kizuna Centrosomal Protein); *IDUA* (Alpha-L-Iduronidase); *PKHD1* (PKHD1 Fibrocystin/Polyductin); *COL10A1* (Collagen Type X Alpha 1 Chain); *ZMPSTE24* (Zinc Metallopeptidase STE24); *FBN1* (Fibrillin 1); *CYBA* (Cytochrome B-245 Alpha Chain); *VCP* (Valosin Containing Protein).

Likely pathogenic alleles were found in: *HCN2* (Hyperpolarization Activated Cyclic Nucleotide Gated Potassium Channel 2); *GALNS* (Galactosamine (N-Acetyl)-6-Sulfatase); *AMPD1* (Adenosine Monophosphate Deaminase 1); *KMT2C* (Lysine Methyltransferase 2C); *CENPJ* (Centromere Protein J); *C8A* (Complement C8 Alpha Chain); *PRKRA* (Protein Kinase, Interferon Inducible Double Stranded RNA Dependent Activator); *CNGA1* (Cyclic Nucleotide Gated Channel Subunit Alpha 1); *GJB2* (Gap Junction Protein Beta 2); *HIBCH* (3-Hydroxyisobutyryl-CoA Hydrolase); *LRP5LD* (L Receptor Related Protein 5); *LEPR* (Leptin Receptor); *LONP1* (Lon Peptidase 1, Mitochondrial); *HMGCS2* (3-Hydroxy-3-Methylglutaryl-CoA Synthase 2); *IHH* (Indian Hedgehog Signaling Molecule); *ANK2* (Ankyrin 2); *RYR1* (Ryanodine Receptor 1); *ITPR2* (Inositol 1,4,5-Trisphosphate Receptor Type 2); *CA12* (Carbonic Anhydrase 12); *UVSSA* (UV Stimulated Scaffold Protein A); VAC14 (Vac14, PIKFYVE Complex Component); *FAM161A* (Family With Sequence Similarity 161 Member A); *NPHP3* (Nephrocystin 3); *KRT5* (Keratin 5); *VCP* (Valosin Containing Protein); *GLDC* (Glycine Decarboxylase).

Supplementary Figure 2. Sequencing depth of all carriers and homozygotes for pathogenic/ likely pathogenic variants.

Pathogenic alleles were found in the following genes: *HFE* (Homeostatic iron regulator); *BTD* (Biotinidase); *FLG* (Filaggrin); *FANCM* (Fanconi Anemia Complementation Group M); *GALNS* (Galactosamine N-Acetyl-6-Sulfatase); *PEX7* (Peroxisomal Biogenesis Factor 7); *ACADM* (Acyl-CoA Dehydrogenase Medium Chain); *LIG4* (DNA Ligase 4); *TACR3* (Tachykinin Receptor 3); *ALPL* (Alkaline Phosphatase, Biomineralization Associated); *C8A* (Complement Component 8 Alpha Chain); *RAG1* (Recombination Activating 1); *GJB2* (Gap Junction Protein Beta 2, Connexin 26); *RDH5* (Retinol Dehydrogenase 5); *EIF2B2* (Eukaryotic Translation Initiation Factor 2B Subunit Beta); *HEXA* (Hexosaminidase Subunit Alpha); *LONP1* (Lon Peptidase 1, Mitochondrial); *HIBCH* (3-Hydroxyisobutyryl-CoA Hydrolase); *KIZ* (Kizuna Centrosomal Protein); *IDUA* (Alpha-L-Iduronidase); *PKHD1* (PKHD1 Fibrocystin/Polyductin); *COL10A1* (Collagen Type X Alpha 1 Chain); *ZMPSTE24* (Zinc Metallopeptidase STE24); *FBN1* (Fibrillin 1); *CYBA* (Cytochrome B-245 Alpha Chain); *VCP* (Valosin Containing Protein).

Likely pathogenic alleles were found in: *HCN2* (Hyperpolarization Activated Cyclic Nucleotide Gated Potassium Channel 2); *GALNS* (Galactosamine (N-Acetyl)-6-Sulfatase); *AMPD1* (Adenosine Monophosphate Deaminase 1); *KMT2C* (Lysine Methyltransferase 2C); *CENPJ* (Centromere Protein J); *C8A* (Complement C8 Alpha Chain); *PRKRA* (Protein Kinase, Interferon Inducible Double Stranded RNA Dependent Activator); *CNGA1* (Cyclic Nucleotide Gated Channel Subunit Alpha 1); *GJB2* (Gap Junction Protein Beta 2); *HIBCH* (3-Hydroxyisobutyryl-CoA Hydrolase); *LRP5LD* (L Receptor Related Protein 5); *LEPR* (Leptin Receptor); *LONP1* (Lon Peptidase 1, Mitochondrial); *HMGCS2* (3-Hydroxy-3-Methylglutaryl-CoA Synthase 2); *IHH* (Indian Hedgehog Signaling Molecule); *ANK2* (Ankyrin 2); *RYR1* (Ryanodine Receptor 1); *ITPR2* (Inositol 1,4,5-Trisphosphate Receptor Type 2); *CA12* (Carbonic Anhydrase 12); *UVSSA* (UV Stimulated Scaffold Protein A); VAC14 (Vac14, PIKFYVE Complex Component); *FAM161A* (Family With Sequence Similarity 161 Member A); *NPHP3* (Nephrocystin 3); *KRT5* (Keratin 5); *VCP* (Valosin Containing Protein); *GLDC* (Glycine Decarboxylase).

Supplementary Table 1. Rare and low frequency likely pathogenic variants found in South-Brazilian Mennonites.

| **Gene** | **dbSNP ID** | | **HGVS cDNA** | | **HGVS protein** | | **Type of mutation** | | **SBM %**  **n=650**  ***(n=278)*** | | **non-FIN EURO % (n)** | | ***p**** | | **Amish % (n)** | | ***p***** | | **ABRAOM % (n)** | | ***p****** | |
| --- | --- | --- | --- | --- | --- | --- | --- | --- | --- | --- | --- | --- | --- | --- | --- | --- | --- | --- | --- | --- | --- | --- |
| *HCN2* | rs1357025725 | c.1055A>T | | - | | Missense, splice region variant | | 3.23  *(5.40)* | | - | | - | | - | | - | | - | | - | |  |
| *GALNS* | rs773283163 | c.769G>A | | p.Ala257Thr | | Missense | | 2.77  *(3.96)* | | 0.001  (1179896) | | **p <0.001**  ***p <0.001*** | | 0.0  (912) | | **p <0.001**  ***p <0.001*** | | - | | - | |  |
| *AMPD1* | rs145328844 | c.1974+1G>A | | - | | Splice donor | | 2.15  *(2.16)* | | 0.01 (1179744) | | **p <0.001**  ***p <0.001*** | | 0.0  (912) | | **p <0.001**  ***p <0.001*** | | - | | - | |  |
| *KMT2C* | rs751158858 | c.1013-2A>G | | - | | Splice acceptor | | 1.69  *(3.96)* | | - | | - | | - | | - | | - | | - | |  |
| *CENPJ* | rs768331750 | c.2693-2A>G | | - | | Splice acceptor | | 1.69  *(2.16)* | | 9 x 10^-4^  (1179960) | | **p <0.001**  ***p <0.001*** | | 0.0  (912) | | **p <0.001**  ***p <0.001*** | | - | | - | |  |
| *C8A* | rs1249209022 | c.454del | | p.Ala152GlnfsTer8 | | Frameshift variant | | 1.54  *(1.80)* | | 6 x 10^-4^  (1179844) | | **p <0.001**  ***p <0.001*** | | 0.0  (912) | | **p <0.001**  ***p <0.001*** | | - | | - | |  |
| *PRKRA* | rs779091550 | c.515-2A>T | | - | | Splice acceptor | | 1.54  (-) | | - | | -  - | | - | | -  - | | -  - | | -  - | |  |
| *CNGA1* | rs539600817 | c.349G>T | | p.Glu117Ter | | Stop gained | | 1.39  *(2.16)* | | - | | - | | - | | - | | - | | - | |  |
| *GJB2* | rs80338945 | c.269T>C | | p.Leu90Pro | | Missense | | 1.39  *(1.08)* | | 0.082  (1180030) | | **p <0.001**  ***0.005*** | | 0.0  (912) | | **p <0.001**  ***0.013*** | | 0.085  (2342) | | **p <0.001**  ***0.013*** | |  |
| *HIBCH* | rs1029330782 | c.518-1G>A | | - | | Splice acceptor | | 1.23  *(1.08)* | | 4 x 10^-4^  (1178416) | | **p <0.001**  ***p <0.001*** | | 0.0  (912) | | **p <0.001**  ***0.012*** | | - | | - | |  |
| *LRP5* | rs761919591 | c.1265C>G | | p.Ala422Gly | | Missense | | 1.23  *(1.80)* | | - | | - | | - | | - | | - | | - | |  |
| *LRP5* | - | c.1253C>G | | p.Pro418Arg | | Missense | | 1.23  *(1.80)* | | - | | - | | - | | - | | - | | - | |  |
| *LEPR* | - | c.2213-2A>G | | - | | Splice acceptor | | 1.23  *(0.72)* | | - | | - | | - | | - | | - | | - | |  |
| *LONP1* | rs879255247 | c.2026C>T | | p.Pro676Ser | | Missense | | 1.23  *(0.72)* | | 8.5 x 10^-4^  (1167556) | | **p <0.001**  ***p <0.001*** | | 0.0  (912) | | **p <0.001**  *0.054* | | - | | - | |  |
| *HMGCS2* | rs777348530 | c.772T>C | | p.Ser258Pro | | Missense | | 1.23  *(1.08)* | | - | | - | | - | | - | | - | | - | |  |
| *IHH* | rs1217838827 | c.367C>T | | p.Pro123Ser | | Missense | | 1.08  *(1.08)* | | 2.5 x 10^-4^  (1180016) | | **p <0.001**  ***p <0.001*** | | 0.0  (910) | | **0.002**  ***0.013*** | | - | | - | |  |
| *ANK2* | - | c.5720C>A | | p.Ser1907Ter | | Stop gained | | 0.92  *(1.08)* | | - | | - | | - | | - | | - | | - | |  |
| *RYR1* | - | c.6797-1G>T | | - | | Splice acceptor | | 0.77  *(1.44)* | | - | | - | | - | | - | | - | | - | |  |
| *ITPR2* | - | c.1895A>T | | p.Asp632Val | | Missense | | 0.77  *(0.36)* | | - | | - | | - | | - | | - | | - | |  |
| *CA12* | - | c.166T>G | | p.Ser56Ala | | Missense | | 0.77  *(0.36)* | | 8.5 x 10^-7^  (1179166) | | **p <0.001**  ***p <0.001*** | | 0.0  (908) | | **0.013**  *0.23* | | - | | - | |  |
| *UVSSA* | - | c.319_320del | | p.Gln107GlufsTer15 | | Frameshift variant | | 0.62  *(1.08)* | | - | | - | | - | | - | | - | | - | |  |
| *VAC14* | rs1555527284 | c.486+3A>G | | - | | Splice donor | | 0.62  *(0.36)* | | - | | - | | - | | - | | - | | - | |  |
| *FAM161A* | - | c.523G>T | | p.Glu175Ter | | Stop gained | | 0.62  *(1.08)* | | - | | - | | - | | - | | - | | - | |  |
| *NPHP3* | - | c.2099del | | p.Leu700GlnfsTer22 | | Frameshift variant | | 0.62  *(1.08)* | | - | | - | | - | | - | | - | | - | |  |
| *KRT5* | rs56790237 | c.982G>C | | p.Asp328His | | Missense | | 0.62  *(0.72)* | | - | | - | | - | | - | | - | | - | |  |
| *VCP* | - | c.2078G>T | | p.Arg693Leu | | Missense | | 0.62  *(1.44)* | | - | | - | | - | | - | | - | | - | |  |
| *GLDC* | rs747853668 | c.1607G>A | | p.Arg536Gln | | Missense | | 0.62  *(0.36)* | | 5 x 10^-4^  (1179128) | | **p <0.001**  ***p <0.001*** | | 0.0  (912) | | **0.03**  *0.23* | | - | | - | |  |

Values in italics were obtained after the exclusion of first-degree relatives. FirstSBM % (n) – Percentage of allele frequencies found in South-Brazilian Mennonites and sample number of alleles; non-FIN EURO % (n) - Percentage of allele frequencies and sample number of alleles reported in the gnomAD database (v4.1, non-Finnish European populations); *p** - p-value calculated with Fisher's exact test to compare allele frequency between South Brazilian Mennonites vs non-Finnish Europeans; Amish % (n) - Percentage of allele frequencies and sample number of alleles reported in the gnomAD database (v4.1, Amish population); *p*** - p-value calculated Fisher's exact test to compare allele frequency between South Brazilian Mennonites and Amish populations; ABraOM % (n) – Percentage of allele frequencies and sample number of alleles reported in ABraOM (database of Brazilian genomic variants), *p**** - p-value calculated with Fisher's exact test to compare allele frequency between South Brazilian Mennonites and Brazilians; all p-values < 0.05 were considered statistically significant; In bold: statistically significant *p*-values; dbSNP ID – Identifier assigned to a single nucleotide polymorphism (SNP) in the Database of Single Nucleotide Polymorphisms; HGVS cDNA – Human Genome Variation Society notation for variants at the complementary DNA (cDNA) level; HGVS Protein – Human Genome Variation Society notation for variants at the protein level. Hyperpolarization Activated Cyclic Nucleotide Gated Potassium Channel 2 (*HCN2*); Galactosamine (N-Acetyl)-6-Sulfatase (*GALNS*); Adenosine Monophosphate Deaminase 1 (*AMPD1*); Lysine Methyltransferase 2C (*KMT2C*); Centromere Protein J (*CENPJ*); Complement C8 Alpha Chain (*C8A*); Protein Kinase, Interferon Inducible Double Stranded RNA Dependent Activator (*PRKRA*); Cyclic Nucleotide Gated Channel Subunit Alpha 1 (*CNGA1*); Gap Junction Protein Beta 2 (*GJB2*); 3-Hydroxyisobutyryl-CoA Hydrolase (*HIBCH*); LDL Receptor Related Protein 5 (*LRP5*); Leptin Receptor (*LEPR*); Lon Peptidase 1, Mitochondrial *(LONP1*); 3-Hydroxy-3-Methylglutaryl-CoA Synthase 2 (*HMGCS2*); Indian Hedgehog Signaling Molecule (*IHH*); Ankyrin 2 (*ANK2*); Ryanodine Receptor 1 (*RYR1*); Inositol 1,4,5-Trisphosphate Receptor Type 2 (ITPR2); Carbonic Anhydrase 12 (*CA12*); UV Stimulated Scaffold Protein A (*UVSSA*); Vac14, PIKFYVE Complex Component (VAC14); Family With Sequence Similarity 161 Member A (*FAM161A*); Nephrocystin 3 (*NPHP3*); Keratin 5 (*KRT5*); Valosin Containing Protein (*VCP*); Glycine Decarboxylase (*GLDC*).

Supplementary Table 2. Heterozygosity values within South Brazilian variant carriers based on surname frequencies.

| **Gene** | **Variant** | **n** | **Frequency** | **Surname heterozygosity** | **Standard Deviation** |
| --- | --- | --- | --- | --- | --- |
| *ANKRD52* | *12_56245596_C_G* | 6 | 0.022 | 0.778 | 0.037 |
| *DDX11* | *rs376819535* | 6 | 0.022 | 0.778 | 0.037 |
| *FAM161A* | *2_61840481_C_A* | 6 | 0.022 | 0.778 | 0.037 |
| *GJB2* | *rs80338945* | 6 | 0.022 | 0.778 | 0.037 |
| *HIBCH* | *rs1029330782* | 6 | 0.022 | 0.778 | 0.037 |
| *HMGCS2* | *rs777348530* | 6 | 0.022 | 0.778 | 0.037 |
| *IHH* | *rs1217838827* | 6 | 0.022 | 0.778 | 0.037 |
| *LIG4* | *rs780879476* | 12 | 0.043 | 0.806 | 0.038 |
| *SIX3* | *rs757457966* | 8 | 0.029 | 0.813 | 0.024 |
| *C8A* | *rs1249209022* | 10 | 0.036 | 0.820 | 0.030 |
| *KLF1* | *19_12885056_C_CCTTG* | 6 | 0.022 | 0.833 | 0.000 |
| *ANK2* | *4_113354338_C_A* | 6 | 0.022 | 0.833 | 0.000 |
| *CFTR* | *rs143486492* | 6 | 0.022 | 0.833 | 0.000 |
| *HPS3* | *rs121908316* | 6 | 0.022 | 0.833 | 0.000 |
| *NPHP3* | *3_132696802_TA_T* | 6 | 0.022 | 0.833 | 0.000 |
| *ACADM* | *rs875989859* | 10 | 0.036 | 0.840 | 0.028 |
| *LRP5* | *11_68386553_C_G* | 10 | 0.036 | 0.840 | 0.016 |
| *PLTP* | *rs1600484803* | 8 | 0.029 | 0.844 | 0.050 |
| *ALPL* | *rs121918009* | 12 | 0.043 | 0.861 | 0.019 |
| *RYR1* | *19_38496859_G_T* | 8 | 0.029 | 0.875 | 0.000 |
| *SBDS* | *rs113993993* | 8 | 0.029 | 0.875 | 0.000 |
| *VCP* | *9_35059146_C_A* | 8 | 0.029 | 0.875 | 0.000 |
| *TACR3* | *rs144292455* | 14 | 0.050 | 0.888 | 0.013 |
| *AMPD1* | *rs145328844* | 12 | 0.043 | 0.889 | 0.009 |
| *CADPS* | *3_62765872_T_G* | 12 | 0.043 | 0.903 | 0.006 |
| *CENPJ* | *rs768331750* | 12 | 0.043 | 0.903 | 0.006 |
| *CNGA1* | *rs539600817* | 12 | 0.043 | 0.903 | 0.006 |
| *RDH5* | *rs62638191* | 12 | 0.043 | 0.903 | 0.006 |
| *ZMPSTE24* | *1_40290870_G_GT* | 6 | 0.022 | 0.906 | 0.000 |
| *ABCA1* | *rs1005990127* | 14 | 0.050 | 0.908 | 0.006 |
| *FANCM* | *rs147021911* | 14 | 0.050 | 0.908 | 0.006 |
| *GALNS* | *rs773283163* | 22 | 0.079 | 0.913 | 0.008 |
| *CLINT1* | *5_157813228_C_A* | 18 | 0.065 | 0.914 | 0.007 |
| *PRKRA* | *rs779091550* | 14 | 0.050 | 0.918 | 0.004 |
| *FLG* | *rs61816761* | 16 | 0.058 | 0.922 | 0.004 |
| *PITPNM3* | *17_6457595_T_A* | 14 | 0.302 | 0.929 | 0.000 |
| *KMT2C* | *rs751158858* | 22 | 0.079 | 0.934 | 0.004 |
| *PEX7* | *rs1805137* | 22 | 0.079 | 0.942 | 0.002 |
| *HCN2* | *rs1983305283* | 30 | 0.108 | 0.947 | 0.002 |
| *HFE* | *rs1800562* | 46 | 0.165 | 0.953 | 0.002 |
| *BTD* | *rs13078881* | 38 | 0.137 | 0.954 | 0.002 |
| *UVSSA* | *4_1349741_GCA_G* | 6 | 0.022 | 0.969 | 0.000 |

*ANKRD52 - ankyrin repeat domain-containing protein 52, DDX11 - DEAD/H-box helicase 11, FAM161A - family with sequence similarity 161, member A, GJB2 - Gap junction protein beta 2, HIBCH - 3-hydroxyisobutyryl-CoA hydrolase, HMGCS2 - 3-hydroxy-3-methylglutaryl-CoA synthase 2, IHH - Indian hedgehog signaling molecule, LIG4 - DNA ligase 4, SIX3 - SIX homeobox 3, C8A – complement C8 alpha chain, KLF1 - KLF transcription factor 1, ANK2 - Ankyrin 2, CFTR - CF transmembrane conductance regulator, HPS3 - HPS3 biogenesis of lysosomal organelles complex 2 subunit 1, NPHP3 - Nephrocystin 3, ACADM - Acyl-CoA dehydrogenase medium chain, LRP5 - LDL receptor related protein 5, PLTP - Phospholipid transfer protein, ALPL - Alkaline phosphatase, biomineralization associated, RYR1 - Ryanodine receptor 1, SBDS - SBDS ribosome maturation factor, VCP -valosin containing protein, TACR3 - Tachykinin receptor 3, AMPD1 - Adenosine monophosphate deaminase 1, CADPS - Calcium dependent secretion activator, CENPJ - Centromere protein J, CNGA1 - Cyclic nucleotide gated channel subunit alpha 1, RDH5 - Retinol dehydrogenase 5, ZMPSTE24 - Zinc metallopeptidase STE24, ABCA1 - ATP binding cassette subfamily A member 1, FANCM - FA complementation group M, GALNS - Galactosamine (N-acetyl)-6-sulfatase, CLINT1 - Clathrin interactor 1, PRKRA - Protein activator of interferon induced protein kinase EIF2AK2, FLG – Filaggrin, PITPNM3 - PITPNM family member 3, KMT2C - Lysine methyltransferase 2C, PEX7 - Peroxisomal biogenesis factor 7, HCN2 - Hyperpolarization-activated, cyclic nucleotide-gated K+ 2, HFE - Homeostatic iron regulator, BTD – Biotinidase, UVSSA - UV stimulated scaffold protein A.*

**Supplementary Table 3.** Consequence of the variant and possible phenotype:

| Symbol | Gene name | dbSNP ID | HGVS cDNA | Type | P/LP | AR/AD | Associated phenotypic condition | Variant information |
| --- | --- | --- | --- | --- | --- | --- | --- | --- |
| CID-11: Endocrine, nutritional or metabolic diseases | | | | | | | | |
| *HFE* | Homeostatic iron regulator | rs1800562 | c.845G>A | Missense | P | AR | Hemochromatosis | The c.845G>A mutation in the *HFE* gene, also known as p.Cys282Tyr, is a missense mutation frequently associated with hereditary hemochromatosis. This condition results in excessive accumulation of iron in the body, affecting organs such as the liver, heart and pancreas, and can lead to cirrhosis, diabetes, heart failure and arthritis. This mutation alters the conformation of the *HFE* protein, affecting its interaction with other proteins involved in regulating iron absorption in the intestine. Patients homozygous for the p.Cys282Tyr mutation have a higher risk of developing the disease.  ICD-11: 5C64.1Y - Hemochromatosis |
| *BTD* | Biotinidase | rs13078881 | c.1270G>C | Missense | P | AR | Biotinidase deficiency | The c.1270G>C mutation in the *BTD* gene, resulting in the amino acid aspartic acid being replaced by histidine at position 424 of the protein (p.Asp424His), is a missense mutation associated with biotinidase deficiency. The *BTD* gene encodes the biotinidase enzyme, which is crucial for the metabolism of biotin, an essential vitamin for various cellular functions, including fatty acid synthesis and amino acid metabolism. Deficiency of this enzyme leads to impaired biotin recycling, resulting in symptoms such as neurological problems, seizures, dermatitis and alopecia, which can be prevented with adequate biotin supplementation.  ICD-11: 4B4Y - Biotinidase deficiency |
| *ALPL* | Alkaline phosphatase, biomineralization associated | rs121918009 | c.1001G>A | Missense | P | AR | Hypophosphatasia | The c.1001G>A (p.Gly334Asp) variant in the *ALPL* gene is a missense mutation that replaces glycine with aspartic acid at position 334 of the alkaline phosphatase protein. This mutation is associated with hypophosphatasia, a rare genetic disease that affects bone mineralization and can vary in severity from severe perinatal forms to less severe adult forms. Studies indicate that this alteration causes a loss of function in the protein, which is essential for the metabolism of phosphates in bone tissue and teeth. Consequently, this loss of function can lead to symptoms such as fragile bones, bone pain, and dental complications.  ICD-11: 5C64.3 - Phosphorus metabolism or phosphatase disorders |
| *GALNS* | Galactosamine (N-acetyl)-6-sulfatase | rs773283163 | c.769G>A | Missense | LP | AR | Mucopolissacaridose, MPS-IV-A | The c.769G>A mutation in the *GALNS* gene, resulting in the amino acid alanine being replaced by threonine (p.Ala257Thr), is a missense mutation. The GALNS gene encodes the galactosamine-6-sulfatase enzyme, which plays a crucial role in the metabolism of glycosaminoglycans (GAGs), especially in the degradation of keratan sulfate and chondroitin sulfate. Deficiencies in the activity of this enzyme lead to a condition known as mucopolysaccharidosis type IV (MPS IV) or Morquio syndrome.  5C56.32 - Mucopolysaccharidosis type 4A |
| *IDUA* | Mucopolysaccharidose type 1 | rs199801029 | c.979G>C | Missense | P | AR | Mucopolysaccharidose  type 1 | The c.979G>C (p.Ala327Pro) variant in the *IDUA* gene is a missense mutation associated with mucopolysaccharidosis type I (MPS I), an autosomal recessive disease caused by the deficiency of the enzyme alpha-L-iduronidase. This enzyme is essential for the metabolism of glycosaminoglycans (GAGs), and its deficiency leads to the accumulation of GAGs in various tissues, causing progressive symptoms such as skeletal problems, heart disease, and neurological impairment.  ICD-11: 5C55.0 - Mucopolysaccharidosis, type 1 |
| *ACADM* | Acyl-CoA dehydrogenase medium chain | rs875989859 | c.347G>A | Missense | P | AR | Medium-chain acyl-coenzyme A dehydrogenase deficiency. | The *ACADM* c.347G>A (p.Cys116Tyr) variant is associated with medium-chain acyl-CoA deficiency (MCAD), a disorder of fatty acid metabolism. This condition is characterized by an inability of the body to properly metabolize medium-chain fatty acids, leading to episodes of hypoglycemia and potentially serious complications such as encephalopathy and sudden death in infants, especially after periods of prolonged fasting or acute illness.  ICD-11: 5C51.0 - Disorders of the pentose phosphate pathway. |
| *LEPR* | Leptin receptor |  | c.2213-2A>G | Splice acceptor | LP | AR | Morbid Obesity | The c.2213-2A>G mutation in the *LEPR* gene is a variant that affects the splice acceptor site, which can compromise the correct processing of messenger RNA and result in a non-functional or absent leptin receptor protein. This mutation has been associated with leptin receptor deficiency, an autosomal recessive condition characterized by severe early-onset obesity due to the inability to regulate satiety. This deficiency can also result in hypogonadotrophic hypogonadism, affecting sexual development.  This variant is classified as “pathogenic” due to its impact on the functioning of leptin, the hormone responsible for regulating appetite and metabolism.  ICD-11: 5B81.0 - Obesity due to energy imbalance |
| *HMGCS2* | 3-hydroxy-3-methylglutaryl-CoA synthase 2 | rs777348530 | c.772T>C | Missense | LP | AR | 3-hydroxy-3-methylglutaryl-CoA synthase (HMG-CoA synthase) deficiency | The c.772T>C (p.Ser258Pro) mutation in the *HMGCS2* gene is a missense alteration associated with mitochondrial HMG-CoA synthase deficiency, a rare metabolic condition that affects the production of ketone bodies. This deficiency interferes with the body's ability to generate energy from lipids, especially during periods of fasting, leading to episodes of hypoglycemia, metabolic acidosis and difficulty responding to metabolic stress.  ICD-11: 5C52.02 - Disorders of ketone body metabolism |
| *HEXA* | Hexosaminidase subunit alpha | rs121907954 | c.805G>A | Missense, splice region variant | P | AR | GM2 Gangliosidosis or Tay-Sachs disease | The missense mutation and splice variant *HEXA* c.805G>A (p.Gly269Ser) is associated with Tay-Sachs disease, a GM2 gangliosidosis that leads to a deficiency of the enzyme beta-hexosaminidase A. This alteration is common in populations of Ashkenazi Jewish origin and is mainly related to the late-onset form of the disease, which manifests in adulthood and can include symptoms such as muscle weakness, ataxia, and progressive neurological impairment. The enzyme deficiency results in the accumulation of GM2 gangliosides in nerve cells, leading to significant cell damage.  ICD-11: 8E71.1 - Tay-Sachs disease |
| *ITPR2* | Inositol 1,4,5-trisphosphate receptor type 2 |  | c.1887-5_1890del | Splice acceptor | LP | AR | Anhidrosis | The c.1887-5_1890del variant in the *ITPR2* gene is a deletion that impacts the splice acceptor site, which is essential for the correct processing of messenger RNA. This type of mutation can lead to loss of protein function due to incorrect splicing, which is important for the function of the inositol 1,4,5-triphosphate receptor type 2 (IP3R2). This receptor plays a central role in intracellular calcium signaling, which affects various cellular processes, including sweating and cellular homeostasis. The mutation is currently classified as probably pathogenic by the ACMG, with indications that it may contribute to anhidrosis in affected individuals.  ICD-11: EE01 Hypohidrosis |
| *GLDC* | Glycine decarboxylase | rs747853668 | c.1607G>A | Missense | LP | AR | Non-ketotic hyperglycinemia (NKH) | A variante c.1607G>A (p.Arg536Gln) no gene *GLDC*, que resulta na substituição de arginina por glutamina na posição 536. Esta mutação é associada à hiperglicinemia não cetótica (GCE), também conhecida como encefalopatia glicínica. Trata-se de um distúrbio metabólico raro que afeta a degradação da glicina, levando a níveis elevados desse aminoácido, especialmente no sistema nervoso central.  A mutação impacta a função da enzima glicina descarboxilase, essencial no sistema de clivagem da glicina. Estudos mostraram que variantes no *GLDC* associadas a essa condição frequentemente comprometem a função da proteína, levando a um acúmulo tóxico de glicina no organismo​.  CID-11: 5C50.1 - doença do metabolismo dos aminoácidos. |
| *AMPD1* | Adenosine Monophosphate deaminase 1 | rs145328844 | c.1974+1G>A | Splice donor | LP | AR | AMPD1 Deficiency Syndrome | The genetic variant c.1974+1G>A in the *AMPD1* gene (adenosine monophosphate deaminase 1), commonly referenced by the ID rs145328844, involves a splice donor alteration that may impact the function of the AMPD1 enzyme. This enzyme plays a critical role in muscle metabolism by regulating adenosine monophosphate (AMP) levels and is essential for energy regeneration in muscle cells. The c.1974+1G>A mutation affects the processing of *AMPD1* gene messenger RNA, potentially resulting in a truncated or absent protein. This dysfunction reduces the muscle's ability to regenerate ATP (adenosine triphosphate), which is essential for muscle contraction.  ICD-11: 5C55 Inborn errors of purine, pyrimidine, or nucleotide metabolism |
| CID-11: Developmental anomalies | | | | | | | | |
| *CENPJ* | Centromere protein J | rs768331750 | c.2693-2A>G | Splice acceptor | P | AR | Seckel syndrome 4 | The c.2693-2A>G variant in the *CENPJ* gene is a mutation that affects the splice acceptor site, which can interfere with the correct removal of introns during messenger RNA processing. The alteration probably leads to a loss of function of the *CENPJ* gene, which is important for cell development and regulation, although this specific mutation is not directly associated with cancer. Studies indicate that pathogenic variants in the *CENPJ* gene are involved in development-related disorders, such as autosomal recessive primary microcephaly.  ICD-11: LA05.0 - Microcephaly |
| *COL10A1* | Collagen type X alpha 1 chain | rs779802963 | c.211C>T | Stop gained | P | AD | Schmid-type metaphyseal chondrodysplasia (SMCD) | A mutação c.211C>T (p.Arg71Ter) no gene *COL10A1* é uma variante de "ganho de parada" (stop gained) que resulta em um códon de terminação prematura, levando à produção de uma proteína truncada e, provavelmente, disfuncional. Este gene codifica o colágeno tipo X, que é essencial para o desenvolvimento adequado da placa de crescimento nos ossos, particularmente durante o crescimento infantil. Esta mutação foi associada à displasia metafisária de Schmid, uma condição caracterizada por baixa estatura, deformidades ósseas e problemas de crescimento.  CID-11: LD24.7 - Displasias metafisárias múltiplas |
| *ZMPSTE24* | zinc metalloproteinase STE24. | rs137854889 | c.1085dup | Frameshift variant | P | AR | Mandibuloacral Dysplasia Type B | The *ZMPSTE24* c.1085dup (p.Leu362PhefsTer19) mutation is a frameshift variant that causes an early termination in protein translation, leading to a loss of function. This mutation is associated with severe conditions such as Restrictive Dermopathy and Mandibuloacral Dysplasia Type B, both related to disorders in the processing of lamin A, a protein essential for nuclear structure and function. Patients with restrictive dermopathy exhibit extremely rigid and thick skin, respiratory complications, and the condition is usually fatal in the neonatal period. In mandibuloacral dysplasia, symptoms include lipodystrophy, premature aging, and skeletal deformities.  ICD-11: LD27.6Z Genetic lipodystrophy, unspecified |
| *MMP13* | Matrix metallopeptidase 13 | rs797044754 | c.772dup | Frameshift variant | P | AR/AD | Spondyloepimetaphyseal dysplasia  Missouri type | The *MMP13* c.772dup mutation (p.Asp258GlyfsTer14) is a frameshift variant that introduces a change in the protein sequence, leading to premature translation arrest. This mutation results in loss of function of the enzyme matrix metalloproteinase 13 (MMP-13), which plays a critical role in collagen degradation and extracellular matrix remodeling. Functional deficiency of MMP-13 is associated with bone conditions such as spondyloepimetaphyseal dysplasia, a rare disorder that affects bone development. Also, deficiency in the AMPD1 enzyme can influence the homeostasis of energy metabolism in chondrocyte cells, contributing to the degeneration of articular cartilage and implicating inflammatory mechanisms that can affect the progression of rheumatoid arthritis, leading to an increase in inflammatory activity in the joints.  ICD-11: LD2B.4 - Spondyloepimetaphyseal dysplasia, FA22 - Polymyalgia rheumatica and FA20 - Rheumatoid polyarthritis |
| *KMT2C* | Lysine-specific methyltransferase 2c | rs751158858 | c.1013-2A>G | Splice acceptor | LP | AR | Kleefstra Syndrome | The mutation in the *KMT2C* gene, identified as a splice acceptor variant (rs751158858) with the alteration c.1013-2A>G in transcript ENST00000262189.11, affects a splicing acceptor site, meaning this substitution interferes with the messenger RNA splicing process. Since the KMT2C gene is essential for epigenetic regulation and chromatin remodeling functions, its mutations can have significant consequences on development and cellular function.  ICD-11: LD2F.1Y Other specified syndromes with multiple structural anomalies, not of environmental origin |
| *IHH* | Indian hedgehog signaling molecule | rs1217838827 | c.367C>T | Missense | LP | AR | Brachydactyly type A1 | The c.367C>T variant in the *IHH* gene results in the substitution of the amino acid proline with serine at position 123 of the protein (p.Pro123Ser). This missense mutation is associated with conditions such as brachydactyly type A1, a congenital malformation characterized by shortening or absence of the middle phalanges of the fingers.  ICD-11: LD26.1 Complex brachydactylies |
| *LRP5* | Low density lipoprotein receptor-related protein 5 | rs761919591 | c.1265C>G | Missense | LP | AD | Familial exudative vitreoretinopathy | The c.1265C>G mutation in the *LRP5* gene results in the substitution of the amino acid alanine with glycine at position 422 of the protein (p.Ala422Gly). The LRP5 gene encodes a protein essential in the Wnt signaling pathway, which is crucial for the development and maintenance of retinal blood vessels. The alanine-to-glycine substitution at position 422 may impair protein function, leading to incomplete or abnormal retinal vascularization, a hallmark of Familial Exudative Vitreoretinopathy (FEVR), an inherited disorder affecting retinal vascular development.  CID-11: 9b80 hereditary vitreoretinal disorders |
| *FBN1* | Fibrillin 1 | rs794728200 | c.3020T>G | Missense | P | AD | Marfan Syndrome | The c.3020T>G variant in the *FBN1* gene results in the substitution of the amino acid leucine with arginine at position 1007 of the fibrillin-1 protein (p.Leu1007Arg). The *FBN1* gene is responsible for encoding fibrillin-1, a glycoprotein essential for the formation of microfibrils in connective tissue, playing a crucial role in the elasticity and integrity of various tissues. Mutations in the FBN1 gene are frequently associated with Marfan Syndrome, a connective tissue disorder that affects multiple systems. However, the specific p.Leu1007Arg variant has a classification of uncertain clinical significance, meaning there is insufficient evidence to directly associate this mutation with specific clinical manifestations.  ICD-11: LD28.01 Marfan Syndrome. |
| *LONP1* | Lon Peptidase 1, Mitochondrial | rs879255247 | c.2026C>T | Missense | LP | AR | CODAS Syndrome | The c.2026C>T mutation in the *LONP1* gene results in the substitution of the amino acid proline with serine at position 676 of the protein (p.Pro676Ser). This missense variant is associated with CODAS Syndrome (Cerebral, Ocular, Dental, Auricular, and Skeletal anomalies), a rare genetic condition characterized by multiple congenital anomalies. Studies have shown that this mutation affects the energy-dependent peptidase activity of the LONP1 protein, impairing the degradation of specific substrates and contributing to the clinical manifestations observed in CODAS Syndrome.  ICD-11: LD2F Syndromes with Multiple Structural Anomalies, Without Predominant Involvement of a Body System |
| CID-11: Diseases of the nervous system | | | | | | | | |
| *EIF2B2* | Eukaryotic translation initiation factor 2B subunit beta | rs113994012 | c.599G>T | Missense, splice region variant | P | AR | Vanishing White Matter Syndrome | The c.599G>T (p.Gly200Val) mutation in the *EIF2B2* gene is a missense variant in the splicing region associated with leukoencephalopathy with white matter disappearance (also known as infantile ataxia syndrome with hypomyelination and white matter disappearance), a neurodegenerative condition that affects the white matter of the central nervous system. This condition is characterized by progressive ataxia, motor problems, and deterioration of neurological function, often diagnosed in childhood. The p.Gly200Val mutation compromises the function of the eukaryotic initiation factor 2B (eIF2B) complex, affecting protein synthesis and the response to cellular stress, which is crucial for maintaining the integrity of the white matter.  ICD-11: 8A46.1 - Leukoencephalopathy with disappearance of white matter |
| *PRKRA* | Protein activator of interferon induced protein kinase EIF2AK2 | rs779091550 | c.515-2A>T | Splice acceptor | LP | AR | Dystonia | The c.515-2A>T mutation in the *PRKRA* gene is an alteration in the splice acceptor site, which can disrupt the proper processing of messenger RNA. This variant has been associated with dystonia type 16 (DYT16), a form of generalized dystonia that causes involuntary muscle contractions and abnormal movements. Mutations in the *PRKRA* gene affect the regulation of cellular stress, and this alteration in particular can lead to abnormal splicing, resulting in loss of function of the protein.  ICD-11: 8A02 Dystonic disorders |
| *HIBCH* | 3-hidroxiisobutiril-CoA hidrolase | rs1029330782 | c.518-1G>A | Splice acceptor | LP | AR | Leigh syndrome | The c.518-1G>A variant in the *HIBCH* gene is a mutation that affects the splice acceptor site, which can lead to incorrect RNA processing and result in the production of a dysfunctional protein. The HIBCH gene is related to valine metabolism and mitochondrial function, and mutations that affect its function are associated with hydroxyisobutyryl-CoA dehydrogenase deficiency (HIBCHD), which can cause a neurodegenerative condition known as Leigh syndrome or Leigh syndrome-like phenotype. This condition manifests itself with severe neurological symptoms such as developmental delay, seizures and respiratory dysfunction.  The loss of function of the *HIBCH* gene due to variants that interrupt splicing is strongly associated with the pathology of HIBCHD. Mutations that affect splicing in genes critical for energy metabolism, such as HIBCH, generally result in severe and early onset symptoms, particularly in conditions related to mitochondrial dysfunction  ICD-11: 5C53.24 Leigh syndrome |
| *VAC14* | VAC14 component of PIKFYVE complex | rs1555527284 | c.486+3A>G | Splice donor | LP | AR | Infantile onset striatonigral degeneration | The *VAC14* c.486+3A>G mutation is a variant that affects the splicing donor site, which can compromise the RNA splicing process and lead to a dysfunctional or missing protein. This variant is associated with infantile onset striatonigral degeneration, a severe neurodegenerative condition characterized by motor problems, spasticity and brain atrophy. The mutation impacts the function of the PIKfyve-VAC14-FIG4 complex, which is essential for intracellular transport and the maintenance of endosomal vesicles  ICD-11: 8A00 - Parkinsonism |
| *HCN2* | Hyperpolarization-activated cyclic nucleotide-gated potassium channel 2; | rs1357025725 | c.1055A>T | Missense, splice region variant | LP | AR | Epilepsy | The genetic variant *HCN2*, associated with the nucleotide alteration c.1055A>T, is a missense variant that affects the splicing region of the *HCN2* gene. This gene is part of the HCN (Hyperpolarization-activated Cyclic Nucleotide-gated channels) family, which are crucial for regulating electrical excitability in cells of the nervous system and the heart. It may be associated with neurological disorders such as epilepsy, chronic neuropathic pain, and other neuronal excitability disorders, as well as cardiac rhythm disturbances like bradyarrhythmia or other arrhythmias.  ICD-11: B Epilepsy |
| *RYR1* | Ryanodine receptor 1 |  | c.6797-1G>T | Splice acceptor | LP | AR/AD | Central core myopathy  (CCD) and Malignant Hyperthermia  (MH) | The c.6797-1G>T mutation in the *RYR1* gene is a variant that affects the splicing acceptor site, which can impair the normal function of the ryanodine receptor 1 (RyR1). This gene is responsible for regulating the flow of calcium in skeletal muscle, playing a crucial role in muscle contraction. Alterations in *RYR1* are known to be associated with a number of congenital myopathies and serious conditions, such as malignant hyperthermia and central core disease, which cause muscle weakness and exercise intolerance. CCD is characterized by muscle weakness from infancy, while malignant hyperthermia poses a potentially fatal risk during anaesthesia, causing a severe hyperthermic response  ICD-11: 8C72.02 - Central core disease and 8C71.0 - Congenital myopathy |
| *CID-11: Diseases of the immune system* | | | | | | | | |
| *C8A* | Complement C8 alpha chain | rs1249209022 | c.454del | Frameshift variant | LP | AR | C8 deficiency, type I | The c.454del variant (p.Ala152GlnfsTer8) in the *C8A* gene is a frameshift mutation that causes the substitution of alanine for glutamine and introduces a premature stop codon after eight amino acids, resulting in a truncated protein. This mutation is associated with a loss of function of the C8A gene, which is part of the complement system and plays a role in the immune response. Mutations that cause loss of function in this gene may be linked to deficiencies in the complement pathway, leading to an increased risk of infections.  ICD-11: 4A00.11 - Immunodeficiency with deficiency of the terminal component of complemente |
| *PEX7* | Peroxisomal biogenesis factor 7 | rs1805137 | c.875T>A | Stop gained | P | AR | Rhizomelic chondrodysplasia punctata type 1. | The c.875T>A mutation in the *PEX7* gene, resulting in the alteration of leucine to a premature stop codon (p.Leu292Ter), is a stop-gained mutation. The *PEX7* gene encodes the peroxin 7 protein, which is essential for transporting proteins to the peroxisomes, cell organelles responsible for oxidizing fatty acids and detoxifying hydrogen peroxide. The peroxin 7 protein is especially important for the import of proteins containing the PTS2 (peroxisomal targeting signal type 2) signaling sequence. The p.Leu292Ter mutation results in the formation of a truncated protein, which is unable to perform its normal function of importing proteins into peroxisomes. Deficiency in the function of the *PEX7* gene is associated with several disorders, including chondrodysplasia rhizomelica punctata type 1 (CDRP1).  ICD-11: 4A01.1 - Combined immune deficiencies, 4A01.10 - Severe combined immunodeficiencies |
| *RAG1* | Recombination activating 1 | rs199474678 | c.1420C>T | Missense | P | AR | Severe Combined Immunodeficiency  (SCID) | The c.1420C>T (p.Arg474Cys) mutation in the *RAG1* gene is a missense variant associated with severe combined immunodeficiency. This condition includes T and B cell deficiency, as well as NK cell expansion, and can manifest with severe and recurrent infections, skin granulomas and other autoimmune manifestations. This mutation results in the exchange of the amino acid arginine for cysteine at position 474, significantly altering the function of the RAG1 protein, which is essential for the recombination of immunoglobulin and T-cell receptor genes. The classification is based on evidence that this variant impairs immune function and is frequently identified in cases of combined immunodeficiency with granulomas.  ICD-11: 4A01.1 - Combined immune deficiencies |
| *LIG4* | DNA ligase 4 | rs780879476 | c.613del | Frameshift variant | P | AR | DNA ligase IV deficiency or LIG4 syndrome | The *LIG4* gene encodes DNA ligase IV, an essential enzyme for DNA repair, particularly in the process of non-homologous end joining (NHEJ). This repair pathway is crucial for fixing double-strand breaks in DNA, a severe type of damage that can lead to mutations and compromise cellular function. Mutations in the *LIG4* gene cause a condition known as DNA ligase IV deficiency or LIG4 syndrome, characterized by immunodeficiency, growth issues, and a predisposition to cancers, especially leukemia and lymphoma.  ICD-11: 4A01.1Y Other specified combined immunodeficiencies |
| *CYBA* | Cytochrome b-245 alpha chain | rs779809359 | c.261C>G | Stop gained | P | AR | Chronic granulomatous disease  (CGD) | The c.261C>G mutation in the *CYBA* gene, which results in the p.Tyr87Ter alteration, is classified as “pathogenic” according to the ACMG criteria. This mutation leads to the production of a premature stop codon, interrupting the synthesis of the CYBA protein, which is essential for the function of the NADPH oxidase enzyme complex. Deficiency of this enzyme causes Chronic Granulomatous Disease (CGD), an inherited condition that affects the immune system's ability to fight infection. Patients with this mutation often have recurrent infections and granulomas due to their neutrophils' inability to produce reactive oxygen species, which are essential for killing pathogens.  ICD-11: 4A40.01 - NADPH oxidase deficiency with chronic granulomatous disease. |
| *VCP* | Valosin-containing protein |  | c.2078G>T | Missense | LP | AD | Inclusion Body Myopathy | The c.2078G>T (p.Arg693Leu) mutation in the *VCP* gene is most directly associated with Inclusion Body Myopathy with Paget Disease and Frontotemporal Dementia (IBMPFD). This condition, caused by mutations in the *VCP* gene, follows an autosomal dominant inheritance pattern and primarily affects skeletal muscles but can also impact the skeletal system and central nervous system to varying degrees.  ICD-11: 4A41.21 Non-inflammatory inclusion body myopathy |
| CID-11: Neoplasms | | | | | | | | |
| *FANCM* | Fanconi anemia, complementation group M | rs147021911 | c.5101C>T | Stop gained | P | AR | Susceptibility to cancer. | The c.5101C>T mutation in the *FANCM* gene, resulting in the substitution of glutamine with a premature stop codon (p.Gln1701Ter), is a stop gained mutation. The *FANCM* gene is part of the DNA repair pathway, known as the FANCD2 repair pathway, which is essential for maintaining genomic stability and responding to DNA damage. Mutations in the FANCM gene are associated with a genetic condition that predisposes individuals to a variety of cancers, including leukemia and solid cancers, due to the body's inability to properly repair DNA breaks. The p.Gln1701Ter alteration results in a truncated protein, which does not have the proper function for which the gene was designed. This can compromise the cells' ability to repair damaged DNA, increasing the risk of mutations and, consequently, the development of cancer.  ICD-11: 2C6Y Other specified malignant neoplasms of breast |
| *KMT2C* | Lysine-specific methyltransferase 2c | rs751158858 | c.1013-2A>G | Splice acceptor | LP | AR | Susceptibility to cancer. | The c.1013-2A>G variant in the *KMT2C* gene (Lysine Methyltransferase 2C), referenced by ID rs751158858, affects a splice acceptor site and may compromise normal gene function. *KMT2C* is a critical gene encoding a lysine methyltransferase, an enzyme essential for regulating gene expression through chromatin modification. This gene participates in histone H3 lysine 4 (H3K4) methylation, a process vital for controlling gene transcription, development, and cell differentiation. Mutations in *KMT2C* have been associated with cancer predisposition due to the gene's critical role in regulating genes involved in cell proliferation and differentiation. |
| *MMP13* | Matrix metallopeptidase 13 | rs797044754 | c.772dup | Frameshift variant | P | AR/AD | Susceptibility to cancer. | The rs797044754 variant (c.772_773insG, p.Asp258GlyfsTer14) is a frameshift mutation in the *MMP13* gene (Matrix Metallopeptidase 13), causing a reading frame shift that generates a premature stop codon 14 amino acids downstream. This frameshift mutation likely leads to a truncated protein. The p.Asp258GlyfsTer14 variant is potentially associated with the development and progression of certain cancers, particularly due to MMP13's role in tumor invasion and metastasis.  ICD-11: Hereditary cancer predisposition syndromes |
| *LIG4* | DNA ligase 4 | rs780879476 | c.613del | Frameshift variant | P | AR | Susceptibility to cancer. | The *LIG4* gene encodes DNA ligase IV, an enzyme essential for DNA repair, particularly in the non-homologous end joining (NHEJ) pathway. This repair pathway is crucial for correcting double-strand DNA breaks, a severe lesion that can lead to mutations and impair cellular function. Mutations in the *LIG4* gene cause a condition known as DNA ligase IV deficiency or LIG4 syndrome, characterized by immunodeficiency, growth issues, and a predisposition to cancers, especially leukemia and lymphoma.  ICD-11: 4A01.1Y Other specified combined immunodeficiencies |
| CID-11: Skin diseases | | | | | | | | |
| *FLG* | Filaggrin | rs61816761 | c.1501C>T | Stop gained | P | AD | Vulgar Ichthyosis and Atopic Dermatitis | The c.1501C>T mutation in the *FLG* gene, known as p.Arg501Ter (or R501X), is a stop-gain variant that causes premature cessation of filaggrin protein production. This mutation is associated with conditions such as ichthyosis vulgaris and atopic dermatitis, both of which are related to defects in the skin barrier. Loss of filaggrin function contributes to the dry, scaly skin characteristic of these diseases, with an increased risk of skin allergies. Studies show that this mutation can be present in both heterozygosity and homozygosity, with the homozygous form generally resulting in a more severe clinical presentation of ichthyosis vulgaris.  ICD-11: EB00.2 - Ichthyosis vulgaris and EA80 Atopic eczema |
| *HPS3* | HPS3 biogenesis of lysosomal organelles complex 2 subunit 1 | rs121908316 | c.1189C>T | Missense | P | AR | Hermansky-Pudlak syndrome 3 (5 cases) | The c.1189C>T (p.Arg397Trp) mutation in the *HPS3* gene is a missense variant associated with Hermansky-Pudlak syndrome type 3 (HPS3), a rare genetic condition that affects pigmentation and blood clotting, as well as being related to lung problems and pulmonary fibrosis. The mutation causes an alteration in the HPS3 protein, impacting the biogenesis of lysosomes and related organelles, which compromises cell function in various tissues, especially the skin, eyes and hematopoietic system. There is strong evidence that the mutation is directly involved in the development of Hermansky-Pudlak syndrome.  ICD-11: 3B81.0 - Albinism with hemorrhagic diathesis |
| *KRT5* | Keratin 5 | rs56790237 | c.982G>C | Missense | LP | AD | Epidermolysis bullosa | The c.982G>C variant in the *KRT5* gene, which results in the amino acid aspartate being replaced by histidine at position 328 (p.Asp328His), lacks consensus and there is limited evidence on the functional impact of this mutation. In terms of frequency, the variant is rare in population databases and there are not enough reports on its direct association with specific diseases.  The KRT5 gene is known to be related to conditions such as epidermolysis bullosa simplex, a skin disorder that affects the integrity of the skin, leading to blisters and skin fragility.  ICD-11: EA84 - Epidermolysis bullosa simplex |
| *ZMPSTE24* | Zinc metalloproteinase ste24 | rs137854889 | c.1085dup | Frameshift variant | P | AR | Restrictive Dermopathy | The *ZMPSTE24* c.1085dup (p.Leu362PhefsTer19) mutation is associated with Restrictive Dermopathy, a rare and severe condition causing premature aging and extreme skin rigidity. This disorder affects the development of connective tissue, resulting in stiff and thick skin that limits growth and breathing. The condition is typically fatal in the neonatal period due to severe respiratory complications. The mutation leads to inadequate production of lamin A, disrupting nuclear organization and causing premature cellular aging. Patients with this condition often present joint stiffness, dry skin, and intrauterine growth restriction, significantly impacting quality of life and survival.  ICD-11: EE6Y Other specified fibromatous diseases of the skin and soft tissue |
| CID-11: Diseases of the visual system | | | | | | | | |
| *FAM161A* | Retinitis pigmentosa 28 |  | c.523G>T | Stop gained | LP | AR | Retinitis pigmentosa 28 | The *FAM161A* gene (family with sequence similarity 161, member A) is involved in various cellular functions, including maintaining the structure and function of the retina. Mutations in this gene are associated with ocular conditions, particularly those resulting in retinal degeneration. Clinical features include night blindness, peripheral visual field loss, and blindness in advanced stages.  ICD-11: 9B71.2 - Hereditary retinal dystrophies |
| *RDH5* | Retinol desidrogenase 5 | rs62638191 | c.712G>T | Missense | P | AR | Fundus albipunctatus | The c.712G>T mutation in the *RDH5* gene, which leads to the substitution of glycine for tryptophan at position 238 (p.Gly238Trp), is a missense alteration associated with retinal pigmentary dystrophy, commonly known as fundus albipunctatus. This autosomal recessive genetic condition affects the ability to adapt to the dark and results in night blindness from childhood. Studies indicate that this variant significantly reduces the activity of the enzyme RDH5, which is essential for the visual cycle of photoreceptors in the retina, interfering with the regeneration of visual pigment.  ICD-11: 9B70 Inherited retinal dystrophies |
| *CNGA1* | Cyclic nucleotide gated channel subunit alpha 1 | rs539600817 | c.349G>T | Stop gained | LP | AR | Retinitis pigmentosa 49 | The c.349G>T (p.Glu117Ter) mutation in the *CNGA1* gene is a "stop gained" variant, resulting in a premature termination codon that disrupts protein synthesis, leading to a truncated and likely non-functional protein. This mutation has been associated with retinitis pigmentosa (RP), specifically the RP49 subtype, a genetic condition causing progressive retinal degeneration and gradual vision loss.  ICD-11: 9B70 - Inherited Retinal Dystrophies |
| *KIZ* | Retinitis pigmentosa 69 | rs587777376 | c.52G>T | Stop gained | P | AR | Retinitis pigmentosa 69 | The c.52G>T (p.Glu18Ter) variant in the *KIZ* gene is a “stop gained” mutation that leads to a premature interruption in the synthesis of the KIZ protein, which is essential for ciliary function and retinal health. This mutation has been associated with retinitis pigmentosa type 69 (RP69), a degenerative eye disease that results in progressive vision loss due to the degeneration of photoreceptor cells. The condition is usually inherited in an autosomal recessive manner.  ICD-11: 9B71.2 - Hereditary retinal dystrophy |
| CID-11: Genitourinary system | | | | | | | | |
| *PKHD1* | PKHD1 ciliary IPT domain containing fibrocystin/polyductin | rs137852944 | c.107C>T | Missense | P | AR | Polycystic kidney disease | The c.107C>T (p.Thr36Met) variant in the *PKHD1* gene is a missense mutation associated with autosomal recessive polycystic kidney disease (ARPKD). This condition is characterized by the formation of cysts in the kidneys and liver, leading to kidney failure and liver complications such as fibrosis. The mutation causes an alteration in the fibrocystin protein, which is essential for the structure and function of the bile ducts and kidneys, resulting in defects in the development of these tissues.  ICD-11: GB40.6 - Autosomal recessive polycystic kidney disease |
| *NPHP3* | Nephrocystin 3 |  |  |  | LP | AR | Nephronophthisis type 3 (or NPHP3) | The *NPHP3* c.2099del (p.Leu700GlnfsTer22) mutation is a frameshift variant that results in the early termination of the nephrocystin-3 protein, associated with Nephronophthisis Type 3. This condition is a form of ciliopathy causing progressive chronic kidney failure, typically manifesting during childhood or adolescence and leading to end-stage renal failure. In addition to kidney involvement, it may be associated with other symptoms, such as liver fibrosis and pancreatic issues.  ICD-11: GB83 - Nephronophthisis |
| *CA12* | Carbonic Anhydrase 12 |  | c.2099del | Frameshift variant | LP | AR | Carbonic anhydrase | The c.166T>G variant in the *CA12* gene results in the substitution of the amino acid serine with alanine at position 56 of the protein (p.Ser56Ala). The CA12 gene encodes the carbonic anhydrase XII enzyme, which plays a crucial role in pH regulation and ion transport in various tissues. Currently, there is insufficient evidence to associate this variant with specific clinical manifestations. Carbonic anhydrase XII is involved in physiological processes, and alterations in its function may potentially impact pH homeostasis and ion transport. However, the clinical significance of this specific mutation remains uncertain.  ICD-11: GB90.44 Renal tubular acidosis |
| CID-11: Diseases of the blood or blood-forming organs | | | | | | | | |
| *SBDS* | SBDS ribosome maturation factor | rs113993993 | c.258+2T>C | Splice donor | P | AR | Shwachman-Diamond syndrome (SDS) | The c.258+2T>C variant in the *SBDS* gene is a mutation that affects the splicing donor site and is associated with Shwachman-Diamond syndrome (SDS), a rare genetic disease inherited in an autosomal recessive manner characterized by pancreatic insufficiency, bone marrow dysfunction and skeletal defects. This mutation is one of the most common pathogenic variants related to SDS and often results in a loss of function of the gene due to the interruption of splicing, which leads to the production of a defective or absent protein.  ICD-11: 3A70.0 Congenital aplastic anemia |
| CID-11: Diseases of the musculoskeletal system or connective tissue | | | | | | | | |
| *MMP13* | Matrix metallopeptidase 13 | rs797044754 | c.772dup | Frameshift variant | P | AR/AD | Arthritis and osteoarthritis | The *MMP13* c.772dup mutation (p.Asp258GlyfsTer14) is a frameshift variant that introduces a change in the protein sequence, leading to premature translation arrest. This mutation results in loss of function of the enzyme matrix metalloproteinase 13 (MMP-13), which plays a critical role in collagen degradation and extracellular matrix remodeling. Functional deficiency of MMP-13 is associated with bone conditions such as spondyloepimetaphyseal dysplasia, a rare disorder that affects bone development. Also, deficiency in the AMPD1 enzyme can influence the homeostasis of energy metabolism in chondrocyte cells, contributing to the degeneration of articular cartilage and implicating inflammatory mechanisms that can affect the progression of rheumatoid arthritis, leading to an increase in inflammatory activity in the joints.  ICD-11: LD2B.4 - Spondyloepimetaphyseal dysplasia, FA22 - Polymyalgia rheumatica and FA20 - Rheumatoid polyarthritis |
| *LRP5* | Low density lipoprotein receptor-related protein 5 | rs761919591 | c.1265C>G | Missense | LP | AD | Early-onset osteoporosis and osteopetrosis | The c.1259G>C and c.1253C>G (p.Pro418Arg) variants in the *LRP5* gene, as well as the c.1265C>G (p.Ala422Gly) variant, are missense mutations that result in amino acid changes in the LRP5 coding region.  LRP5 is an essential gene in the Wnt signaling pathway, which regulates bone development and mineral density, as well as playing roles in the formation and maintenance of ocular structures. Mutations in *LRP5* are associated with various clinical conditions primarily affecting the skeleton and vision.  ICD-11: FB83.1 Osteoporosis and LD24.10 Osteopetrosis |
| CID-11: Diseases of the circulatory system | | | | | | | | |
| *ANK2* | Ankyrin 2 |  | c.5720C>A | Stop gained | LP | AD | QT syndrome 4 and cardiac arrhythmia | The c.5720C>A (p.Ser1907Ter) mutation in the *ANK2* gene is a “stop gained,” variant that results in a premature termination codon, leading to a truncated protein. This gene is responsible for encoding the ankyrin-B protein, which plays an essential role in maintaining the stability and organization of ion channels and transporters in the cell membrane, especially in the heart and brain. Loss-of-function mutations in *ANK2*, such as this one, are associated with various cardiac conditions, including arrhythmias, long QT syndrome, and cardiac conduction dysfunctions.  ICD-11: The ICD-11 classifies this condition under code BA80.0 (Long QT syndrome) for related arrhythmias and 8A42 for other cardiac conduction dysfunctions associated with genetic variants |
| CID-11: Diseases of the ear or mastoid process | | | | | | | | |
| *GJB2* | Gap junction protein beta 2 | rs80338945 | c.269T>C | Missense | LP | AR | Non-Syndromic Congenital Deafness | The c.269T>C (p.Leu90Pro) variant in the *GJB2* gene is a missense mutation that has been widely associated with non-syndromic sensorineural hearing loss, a genetic condition characterized by early onset deafness. This mutation leads to an alteration in the protein connexin 26, which is essential for the formation of gap junction channels between cells. Studies indicate that the alteration affects the functionality of these channels, impairing cell communication in the inner ear and resulting in hearing loss.  Hearing loss caused by this mutation normally occurs in an autosomal recessive inheritance pattern. The p.Leu90Pro mutation is often found in compound heterozygosity with another known mutation in *GJB2*, such as c.35delG, which is also associated with hereditary deafness  ICD-11: AB56 - Hereditary hearing loss |
| CID-11: Conditions related to sexual health | | | | | | | | |
| *TACR3* | Tachykinin receptor 3 | rs144292455 | c.824G>A | Stop gained | P | AR | Congenital hypogonadotrophic hypogonadism  (CHH) | A variante *TACR3* c.824G>A (p.Trp275Ter) é uma mutação que resulta em um códon de parada prematuro no gene TACR3, que codifica o receptor da taquicinina 3. Essa mutação tem sido associada à deficiência isolada de hormônio liberador de gonadotrofina (GnRH), uma condição que pode levar à hipogonadismo em indivíduos afetados​.  CID-11: 5A61.0 Hipopituitarismo |
| CID-11: Diseases of the circulatory system | | | | | | | | |
| *HCN2* | Hyperpolarization Activated Cyclic Nucleotide Gated Potassium And Sodium Channel 2 | rs1357025725 | c.1055A>T | Missense, splice region variant | LP | AR | Sinus bradycardia | The *HCN2* genetic variant, associated with the c.1055A>T nucleotide alteration, is a missense variant that affects the splicing region of the *HCN2* gene. This gene belongs to the family of HCN (Hyperpolarization-activated Cyclic Nucleotide-gated) channels, which are crucial for regulating electrical excitability in the nervous system and heart cells. It may be associated with neurological disorders such as epilepsy, chronic neuropathic pain, and other neuronal excitability disorders, as well as cardiac rhythm disorders, including bradyarrhythmia or other arrhythmias.  ICD-11: BC80.1 Sinus bradycardia |
| CID-11: Symptoms, signs, or clinical findings not elsewhere classified | | | | | | | | |
| *UVSSA* | UV stimulated scaffold protein A |  | c.319_320del | Frameshift variant | LP | AR | UV-sensitive syndrome (UVSS) | The *UVSSA* gene is involved in the transcription-coupled nucleotide excision repair (TC-NER) process, which is essential for correcting DNA damage induced by ultraviolet (UV) radiation. Mutations in this gene are associated with Cockayne syndrome type B (UVSSB) or UV-sensitive syndrome (UVSS), an autosomal recessive condition.  ICD-11: LD2B Syndromes with premature aging as a key feature and ME66.0 Photosensitivity |
